# Assessing the Threat of Amphibian Chytrid Fungus in the Albertine Rift: Past, Present and Future

**DOI:** 10.1371/journal.pone.0145841

**Published:** 2015-12-28

**Authors:** Tracie A. Seimon, Samuel Ayebare, Robert Sekisambu, Emmanuel Muhindo, Guillain Mitamba, Eli Greenbaum, Michele Menegon, Fabio Pupin, Denise McAloose, Alyssa Ammazzalorso, Danny Meirte, Wilbur Lukwago, Mathias Behangana, Anton Seimon, Andrew J. Plumptre

**Affiliations:** 1 Wildlife Conservation Society, Bronx, NY, United States of America; 2 Department of Environmental Sciences, Makerere University, Kampala, Uganda; 3 Department of Biological Sciences, University of Texas at El Paso, El Paso, TX, United States of America; 4 Tropical Biodiversity Section, MUSE, The Science Museum of Trento, Trento, Italy; 5 Department of African Biology, Royal Museum for Central Africa, Tervuren, Belgium; University of South Dakota, UNITED STATES

## Abstract

*Batrachochytrium dendrobatidis* (*Bd*), the cause of chytridiomycosis, is a pathogenic fungus that is found worldwide and is a major contributor to amphibian declines and extinctions. We report results of a comprehensive effort to assess the distribution and threat of *Bd* in one of the Earth’s most important biodiversity hotspots, the Albertine Rift in central Africa. In herpetological surveys conducted between 2010 and 2014, 1018 skin swabs from 17 amphibian genera in 39 sites across the Albertine Rift were tested for *Bd* by PCR. Overall, 19.5% of amphibians tested positive from all sites combined. Skin tissue samples from 163 amphibians were examined histologically; of these two had superficial epidermal intracorneal fungal colonization and lesions consistent with the disease chytridiomycosis. One amphibian was found dead during the surveys, and all others encountered appeared healthy. We found no evidence for *Bd*-induced mortality events, a finding consistent with other studies. To gain a historical perspective about *Bd* in the Albertine Rift, skin swabs from 232 museum-archived amphibians collected as voucher specimens from 1925–1994 were tested for *Bd*. Of these, one sample was positive; an Itombwe River frog (*Phrynobatrachus asper*) collected in 1950 in the Itombwe highlands. This finding represents the earliest record of *Bd* in the Democratic Republic of Congo. We modeled the distribution of *Bd* in the Albertine Rift using MaxEnt software, and trained our model for improved predictability. Our model predicts that *Bd* is currently widespread across the Albertine Rift, with moderate habitat suitability extending into the lowlands. Under climatic modeling scenarios our model predicts that optimal habitat suitability of *Bd* will decrease causing a major range contraction of the fungus by 2080. Our baseline data and modeling predictions are important for comparative studies, especially if significant changes in amphibian health status or climactic conditions are encountered in the future.

## Introduction

The Albertine Rift region in central Africa is a hotspot for biodiversity, the richest area for vertebrates in Africa, and one of the most threatened [1,2]. Intensive agriculture, land and resource pressures, and high rates of habitat loss and land conversion make it a high priority area for conservation [1]. The region, which extends across parts of western Uganda, Rwanda, Burundi and eastern Democratic Republic of Congo (DRC), contains more than 145, or approximately 23%, of all known amphibian species in Africa. At least 42 of these species (29%) are endemic to the Albertine Rift and work over the past decade has led to the discovery of several new species [3–7]. At least 13 amphibian species in the Rift are listed as Threatened by the International Union for the Conservation of Nature (IUCN), 27 are listed as Data Deficient, and 8 species have not been classified, and are likely data deficient or threatened (Plumptre, unpublished data).

Studies focusing on the distribution and abundance of amphibians have identified that *Batrachochytrium dendrobatidis* (*Bd*), alternatively called chytrid, is responsible for the apparent decline and extinction of amphibians in many parts of the world [8–14]. *Bd*, a fungal pathogen that causes chytridiomycosis, is considered to be one of the greatest infectious disease threats to ever face any taxonomic group [12]. *Bd* is associated with infections, die-offs or extinctions in more than 200 amphibian species [14] and is found on every continent except Antarctica [15]. On the African continent, *Bd* has been identified in Ethiopia [16], Tanzania [17], Nigeria [18,19], Kenya [20], Cameroon [21,22], South Africa [23], Gabon [24], and most recently in Madagascar [25]. Environmental niche modeling has predicted widespread occurance of *Bd* in the Albertine Rift [26–28], and to date this fungus has been detected in Kibale Forest in Uganda, [29], the eastern DRC including the Katanga province, Itombwe Natural Reserve, and Kahuzi-Biega National Park [30–32]. The earliest known *Bd*-positive amphibian from the Albertine Rift is a specimen collected in Uganda in 1934 [33]. However, despite recent PCR testing for *Bd* and its known presence in the region, little information is available about whether Albertine Rift amphibians have historically or currently develop the disease chytridiomycosis [30].

In addition to natural spread or anthropogenic introductions of *Bd*, both of which are documented as contributors to infection and disease transmission, climate change is a factor that may impact the presence, persistence, and transmission patterns of *Bd* [15,27,34]. A changing climate can significantly alter or or provide new environmental niches for amphibians, other plant and animal species, and microbial pathogens. For example, it is known that amphibians that inhabit high elevations adapt to climatic warming by upward expansion of their range into new habitat [35,36]. As climate change affects host and pathogen range contraction or expansion, it may also result in new pathogen-host interactions or transmission of *Bd* from carrier to naïve hosts that in either case, can lead to disease emergence [35–37]. Additionally, there is evidence that *Bd* outbreaks occur more frequently when the environment cycles to drier conditions [38,39], as pools become smaller, streamflow is reduced and amphibians are likely to cluster in higher numbers around fewer and diminished water sources that have increased concentrations of infective, fungal zoospores [38]. In the Albertine Rift, climatic models predict significant temperature and precipitation increases by the end of the 21^st^ century [40–42], and recent climate models predict that the worldwide habitat suitability and overall risk of *Bd* infection could be diminished rather than increased by anthropogenic climate change [27]. However, interpretions of species distribution models are complex, and additional factors should be taken into consideration that influence the epidemiology of *Bd* such as the virulence of the pathogen or strain type, the host response to the particular strain of *Bd*, and whether there has been any coevolution of the amphibian species with *Bd* [43].

The main objectives of this study were to document the current distribution and prevalence of chytrid fungus in amphibians in existing and proposed protected areas of the Albertine Rift, determine whether fungal presence is associated with chytridiomycosis, and predict what the effect of climate change will be on *Bd* distribution. We mapped areas of potential *Bd* distribution using data collected during field surveys between 2010 and 2012 and MaxEnt modeling software. We then tested our model using additional data collected in 2013–14 and used this information to retrain the model. We also identified which species are infected by *Bd*, and determined if any had histologic evidence of chytridiomycosis. Focusing on the ITS1-5.8S-ITS2 region of the *Bd* genome, we determined if DNA sequence variation exists within or between protected areas, and looked for *Bd* presence in archived, whole animal voucher specimens from museum collections dating back to 1925. Finally, we used our distribution model to predict the habitat suitability and future distribution of *Bd* over the next century.

## Materials and Methods

### Ethics statement

All work complies with guidelines for the use of live amphibians in field research by the American Society of Ichthyologists and Herpetologists, the Society for the study of Amphibians and Reptiles and The Herpetologists’ League. Swab samples were collected non-invasively from the skin of amphibians that were encountered in the environment and animals were released shortly after handling. Recommended biosecurity practices for minimizing risk of disease transmission between animals and field sites were followed at all sites where amphibian swab samples were collected [44]. No animals were sacrificed specifically for this project. In some cases both swab and tissue samples were taken from voucher amphibian specimens previously collected in the DRC under a separate and unrelated project. IACUC approval for this project was not obtained because WCS institutional requirements for IACUC review do not include field projects that take place outside of our facilities, however non-invasive skin swabbing, capture and release of wild amphibians, collecting dead animals found in the environment, or sampling after euthanasia are all standard techniques and procedures for clinical and pathology examinations or investigations. Trained herpetologists were primarily responsible for collecting swabs from the animals. Sample collection and export permits were obtained from the Royal Museum of Central Africa, Belgium; Ugandan Wildlife Authority and Uganda National Council for Science and Technology, and Makerere University, Uganda; Institut Congolais Pour la Conservation de la Nature (ICCN), DRC; and the Rwandan Development Board, Rwanda (Permits: B2014–001 (Belgium), NS378 (Uganda), and Nos. 31/RDB–T&C/V.U/10 (Rwanda), 02/ICCN/PNKB2011, 01/ICCN/PKNP/2012, 120/ICCN/PKNP/2012, 02/ICCN/PKNP/2013, 074/ICCN/PKNP/2013, 3/ICCN/PKNB/2014, 7/ICCN/PNKB/2014 (DRC). IACUC approval, review of, and approval of sampling procedures were not required by the government agencies for obtaining the sample collection and field research permits. Field surveys were conducted in accordance with the Declining Amphibian Task Force Fieldwork Code of Practice (http://www.amphibianark.org/pdf/Husbandry/The%20DAPTF%20Fieldwork%20Code%20of%20Practice.pdf) and previously described methods [44,45].

### Sample collection and storage

Field expeditions were conducted in Nyungwe Forest National Park in Rwanda from 1–10 December, 2010 and 5–8 March, 2011; in Bwindi Impenetrable Forest National Park, Uganda from 2 January–19 March, 2011; in Kahuzi-Biega National Park, DRC from 11–21 November, 2011; in Itombwe massif, DRC from 13–28 May and 9–15 August, 2012; in Misotshi-Kobogo massif, Ngamikka Park, DRC from 29 October–19 November, 2012; in Itombwe–Mwana, DRC from 18–27 March, 2013; in Kisimba Ikobo Community Reserve, DRC from 14 April, 2013; in Reserve des Gorillas de Punia (Punia Gorilla Reserve)–west of Kahuzi-Biega National Park, DRC from 28 August–17 September, 2013; in lowland Kahuzi-Biega National Park–Nzovu, DRC from 3–27 September, 2013; in lowland Kahuzi Biega Park-Kasese and Itebero, DRC from 5–27 December, 2013; and in the North Balala Forest, DRC from 24 February–15 March, 2014. Herpetological surveys were cut short in Kisimba-Ikobo Reserve, west of Lake Edward in DRC due to insecurity occurring in the area. Therefore very few samples were obtained from this area for *Bd* testing.

Swab samples were collected using BBL 1/8” diameter sterile rayon tipped culture swabs (Fisher Scientific, Hampton NH). Each frog was swabbed 4–5 times each on the underside of the hind feet, thighs, abdomen, and forefeet. Swab samples were dried and stored in airtight plastic containers at room temperature. Handlers changed non-powdered latex or nitrile gloves, or washed hands with soap and water (Nyungwe surveys only) between each animal to prevent contamination. Dead animals that were encountered were collected for chytrid analysis and stored in 70% ethanol. Amphibians were identified to genus or genus and species level during surveys and were released, except for a subset of 163 that were collected as voucher specimens for additional taxonomic identification and histological analysis. A sample of skin from voucher specimens, which had also been swabbed for *Bd* PCR, was collected as either a whole foot or sections from the thigh and preserved in 70% ethanol. Tissue samples were held at room temperature until export for *Bd* testing and histologic processing.

Skin swab samples were also collected from voucher specimens from the Albertine Rift housed in the collections of the Royal Museum for Central Africa (RMCA) in Tervuren, Belgium and Markerere University, Uganda. Amphibians at the RMCA were collected between 1926–1951 by G.F. de Witte, R. F. Laurent, and H. Schouteden. Those from Makerere University were collected by J.B. Goodman, D.F. Oren, M. Behangana and W. Lukwago between 1965–2013. Historical notes on the RMCA voucher specimens indicate that samples from the de Witte collection were preserved in ~5% neutral buffered formalin, while samples from the Laurent and Schouteden collections were also preserved in formalin, however details on the percentage of formaldehyde, or if it was buffered, are not known. Amphibians from these collections were transitioned from formalin to ethanol upon arrival to the RMCA in the following years: Laurent (I.R.S.A.C.) specimens, 1951–1953; Schouteden specimens, 1925, de Witte specimens, 1941. Historical notes on preservation methods in the Makerere University voucher specimens are presented in S2 Table. Gloves were worn and changed between handling individuals from these collections, and when swabbing these specimens for this study to avoid potential cross contamination. However, the herpetologists that collected samples between 1926–2013 for the RMCA and Makerere collections did not wear gloves. Thus our *Bd* prevalence, particularily regarding samples collected from the Bonongo Forest in 2013, may be overestimated because of the possibility of false positives.

### Sampled taxa

Skin swab and/or skin samples were collected from the following genera: Caeciliidae, *Boulengerula*; Dicroglossinae, *Hoplobatrachus*; Hyperoliidae, *Afrixalus spp*., *Hyperolius spp*., *Kassina spp*., *Phlyctimantis spp*.; Pyxicephalidae, *Amietia spp*.; Ranidae, *Hylarana (Amnirana) spp*.; Bufonidae, *Amietophrynus sp*.; Arthroleptidae, *Arthroleptis spp*., *Cardioglossa spp*., *Leptopelis spp*.; Hemisotidae, *Hemisus spp*.; Rhacophorinae, *Chiromantis spp*., Phrynobatrachidae, *Phrynobatrachus spp*.; Ptychadenidae, *Ptychadena spp*.; Pipidae, *Xenopus spp*. In addition, we collected samples from the following species classified as Vulnerable to extinction by the IUCN: *Hyperolius constellatus* (formerly *Hyperolius castaneus*) [46] and *Phrynobatrachus versicolor*.

### Histology

Tissue samples from dead amphibians were preserved in 70% ethanol for histologic examination. Following ethanol fixation, skin samples (n = 163 voucher specimens) and multiple tissues, including skin, from one frog found dead during the current surveys, were processed routinely, paraffin-embedded, sectioned at 5μm, stained with hematoxylin and eosin, and examined by a certified pathologist. A diagnosis of chytridiomycosis was based on identification of characteristic changes associated with the disease, including marked thickening of the epidermis and stratum corneum (epidermal hyperplasia and hyperkeratosis, respectively) and numerous intracorneal chytrid fungal thalli.

### PCR testing

#### PCR testing of field samples

Skin swab samples were air dried and stored in individual sterile cryovial tubes (Fisher Scientific, Hampton, NH, USA) at room temperature. For *Bd* analysis, DNA was extracted using 150 μl of PrepMan (Life Technologies; Grand Island, NY, USA) and extracts were diluted 1:10 in RNAse/DNAse-free water. The samples were then analyzed by real-time quantitative PCR amplification of the internal transcribed spacer (ITS1) and 5.8S rDNA region using established methods [47]. Taqman PCR assays were conducted using a Bio-Rad Mini-Opticon Real–Time PCR detection system. Reaction tubes contained a total of 20 μl consisting of 10 μl of 2X Taqman Environmental Master Mix (Life Technologies), 900 nM of each primer (ITS–1 Chytr3 and 5.8S Chytr), 250 nM of Chytr MGB TaqMan probe (Life Technologies), 2.5 μl of 10X exogenous internal positive control primers and probe, 0.5 μl of 50X exogenous internal positive control DNA, (TaqMan Exogenous Internal Positive Control kit; Life Technologies), and DNase/RNase–free water containing 5 μl of diluted DNA. The exogenous internal positive control reagents served as inhibition controls in the PCR reactions. PCR amplification conditions were: 2 minutes at 50°C, 10 minutes at 95°C, followed by 50 cycles of 15 seconds at 95°C and 1 minute at 60°C. Purified genomic *Bd* DNA or *Bd* plasmid carrying the ITS1–5.8S–ITS2 region was provided by Dr. Allan Pessier (San Diego Institute for Conservation Research, CA) and was diluted to a range of concentrations to generate a standard curve for quantifying copy number in all samples and for use as a positive control. Each sample was run in triplicate. The copy number per swab was calculated by taking the copy number obtained in each triplicate PCR reaction from the standard curve, and then multiplying that number times by its relative proportion of the DNA extract, and then multiplying by the dilution factor. The calculated copy numbers/swab were then averaged and graphed with the standard deviations. Confidence limits for prevalence were calculated based on the Wilson score interval [48].

#### PCR testing of historical specimens

Collection of skin swabs from historical voucher animals was conducted using a modified version of previous methods [49]. Amphibians were placed onto a clean absorbent pad using clean gloved hands. Using a 3.2 mm–6.0 mm tapered brush (Oral B; http://www.oralb.com) clamped to a curved hemostat, the plantar surface of the hind and palmar surface of the fore feet, the thighs, ventral abdomen, pelvis and dorsal part of the body were stroked (each part 4–5 times). The brush was then placed in a sterile 2 ml labeled tube and capped. The same process was then repeated using a sterile swab to collect any remaining loose cells that were not taken up by the brush. The swab was then placed in the tube with the corresponding brush. DNA extraction was performed on the brush and swab together (using the QIAmp DNA minikit, Qiagen Inc., Valencia, CA, USA) using the manufacturer’s protocol with an additional incubation step of 90°C for 1 hour to reverse DNA crosslinking caused by formalin fixation, which was conducted immediately after the addition of AL buffer and proteinase K/56°C incubation step. Prepman (Life Technologies) was used for extraction of DNA from historical swab samples that were stored in ethanol and had no prior formalin fixation.

All samples collected from the RMCA collection were tested for *Bd* in triplicate with internal inhibition controls. For the samples collected from Makerere University, samples were initially tested for *Bd* in singlicate, and any positives were retested in triplicate. All positives from historical samples were retested using 0.2 μl of AmpErase per reaction (UNG, Life Technologies), which was added to the PCR reaction to prevent amplification of contaminating PCR product that could potentially lead to false positives. Because DNA degradation was a concern, an amplification control for the amphibian mitochondrial 16S (SSU rRNA) gene was also run for each sample. For amplification of the 16S, we developed consensus primers (Amph16S–F, GGGATAACAGCGCAATCWAYTT and Amph16S–R, CCCYGATCCAACATCGAGGTCG) to a conserved 72 bp region chosen from a region in an alignment that would broadly amplify the following African species that were represented in Genbank: AF215430 (*Afrixalus laevis*), KC756288 (*Amietia angolensis*), HM770015 (*Amietophrynus regularis*), HQ882846 (*Amietophrynus superciliaris*), DQ283237 (*Arthroleptis schubotzi*), FJ151072 (*Cardioglossa elegans*), GU444000 (*Hyperolius castaneus*), KF447811 (*Hyperolius cinnamomeoventris*), KF562041 (*Hyperolius discodactylus*), KF447815 (*Hyperolius kivuensis*), FJ594084 (*Hyperolius marmoratus*), HQ130782 (*Leptopelis karissimbensis*), HQ130767 (*Leptopelis kivuensis*), FJ829265 (*Phrynobatrachus graueri*), FJ829318 (*Phrynobatrachus versicolor*), and HQ225699 (*Xenopus wittei*). Samples tested for 16S were run in singlicate under the following cycling conditions: 95°C for 5 minutes, followed by 45 cycles of 95°C for 45 seconds, 55°C for 30 seconds, and 72°C for 45 seconds, and a final elongation step of 72°C for 5 minutes. Samples were analyzed using SYBR green on a 2% agarose gel by electrophoresis.

### DNA sequence analysis

Samples positive for *Bd* by quantitative real-time PCR were retested using conventional PCR using primers for the ITS1-5.8S-ITS2 region as previously described [50]. Positive bands were purified using ExoSAP-IT (Affymetrix; Santa Clara, CA, USA) and sequenced in both the forward and reverse directions (Genewiz, Inc. South Plainfield, NJ). Sequences were analyzed, trimmed and aligned using Geneious software (Geneious Pro 6.0, Biomatters LTD. Auckland NZ) and sequence comparisons were performed using the GenBank database and BLASTn. Sequences with >98% identity to those in Genbank were used in the comparative DNA sequence alignments.

### Modeling and statistical analysis

**Fig 1:**
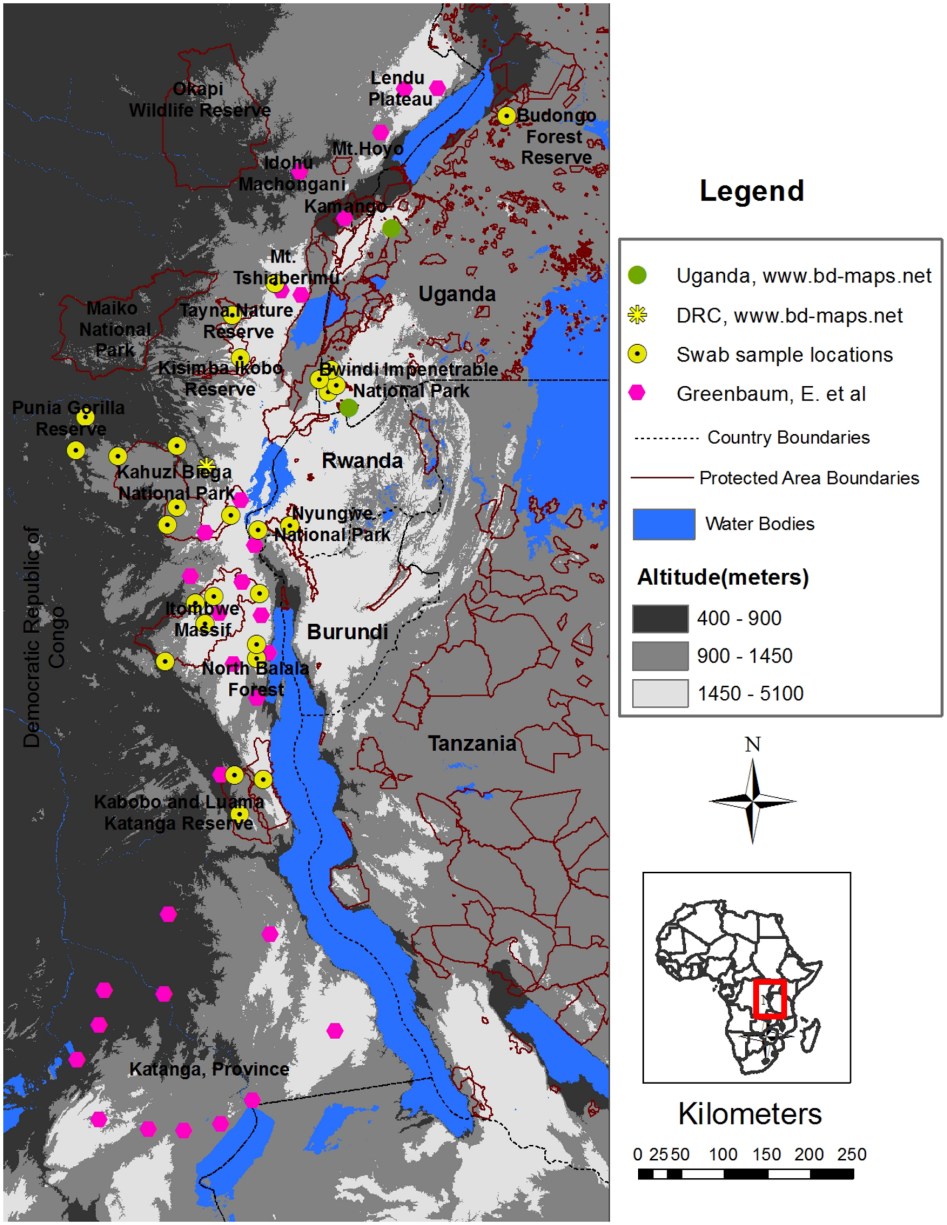
Map of the Albertine Rift region showing the study sites where amphibian skin swab samples have been collected for PCR analysis of *Bd*. Water bodies, country boundaries, protected area boundaries and elevation brackets (meters above sea level) are shown. Yellow circles mark the locations of the sites where samples were collected for this study. The yellow asterick and green circles mark 3 *Bd*-positive sample locations previously identified in DRC and Uganda respectively (data obtained from Bd-maps.net), and the purple hexagons mark sites where samples have been collected from and tested for *Bd* from previous studies [30–32].

Species Distribution Modeling was performed using MaxEnt (MaxEnt version 3.3.3e) [51,52]. MaxEnt is a machine learning modeling technique that requires presence-only occurrence records and has been shown to perform as well or better than other species distribution modeling techniques [51,53,54]. The MaxEnt logarithm estimates the probability distribution with the maximum entropy (i.e., that is most spread out, or closest to uniform), subject to constraints imposed by the information regarding presence records and the background sample across the study area [51,53]. For our modeling approach, a site is defined as a 1km^2^ area. For the initial modeling we used 40 presence-only locations from Kahuzi-Biega National Park, Itombwe, Ngamikka, Nyungwe National Park and Bwindi Impenetrable National Park and a datapoint from 1 site in the DRC taken from Bd-maps.net (accessed October 2014). We then tested the model on 48 new presence-only locations focusing on the northern part of the Albertine Rift and the lowlands. Data to test our model were derived from 6 field expeditions were that were conducted in 2013, in Itombwe and North Balala Forest, Kisimba-Ikobo Community Reserve, Punia Gorilla Reserve, lowland Kahuzi-Biega National Park, and Budongo Forest, and presence-only localities obtained from Eli Greenbaum [30–32], and 2 datapoints from Uganda (obtained from Bd-maps.net: accessed October 2014). Default MaxEnt model parameter settings (auto features, convergence threshold of 0.00001, maximum number of background points = 10,000, regularization multiplier = 1) were used [52]. Models were fitted using 100 bootstrap model runs with 70/30 partition percentage for the training/testing of the data sets accordingly. We assessed the predictive performance of the MaxEnt models with the receiver operating characteristic (ROC) plots [55,56]. A logistic format output was used that produces continuous, linear-scaled maps giving an index of habitat suitability, and hence potential distribution of *Bd*, which range from 0 (unsuitable) to 1 (highly suitable). Model validation was performed using the area under the curve (AUC), and the average training ROC plots AUC for the 100 bootstrap replicate runs was 0.941, with a standard deviation of 0.009. A MaxEnt logistic format output that estimates an index of habitat suitability was used to quantify the risk of *Bd* infection in amphibians. Maps were generated using ArcGIS software developed by ESRI (Environmental Systems Resource Institute). Redlands, California (www.esri.com). The following Shuttle Radar Topography Mission (SRTM; USGS, 2006) Digital Elevation Model (DEM), was sourced from the Global Land Cover Facility, (www.landcover.org) and used as a background in Fig 1: USGS (2006), Shuttle Radar Topography Mission, 3 Arc Second scene Africa, “Filled Finished 2.0, Global Land Cover Facility, University of Maryland, College Park, Maryland, February 2000.

#### Predictor variables

A total of 19 bioclimatic variables at a spatial resolution of ~1 km^2^ were obtained from the WorldClim database (http://www.worldclim.org) [57]. The bioclimatic variables are derived from monthly temperature and rainfall values to produce biologically meaningful variables. Pairwise Pearson correlations between bioclimatic variables were calculated, and only variables with less than (+/-0.75) correlation were retained. For the final model, we used seven variables that are known to have a limiting effect on the distribution of chytrid fungus in the MaxEnt model parameterization, as reported in previous studies [9,23,26–28,58,59]. The covariates used for modeling the distribution of chytrid fungus in the Albertine Rift are as follows: Bio2, Mean diurnal temperature range; Bio5, Maximum temperature of warmest month; Bio6, Minimum temperature of coldest month; Bio7, Annual temperature range; Bio12, Annual precipitation; Bio16, Precipitation of wettest quarter, and Bio17, Precipitation of driest quarter.

#### Estimation of *Bd* occurrence in 2080

To estimate future distributional areas in the Albertine Rift where amphibians are likely to be at risk for *Bd*, output from three General Circulation Models for the year 2080 under the A2a anthropogenic climate change scenario were used in the MaxEnt modeling; 1. CCCMA: CGCM2, from the Canadian Centre for Climate Modeling and Analysis, 2. CSIRO: MK2, from the Commonwealth Scientific and Industrial Research Organization, and 3. HADCM3, from the Hadley Centre for Climate Prediction and Research. The Special Report on Emissions Scenarios describes images of the future using four storylines (A1, A2, B1 and B2) in relation to a wide range of demographic, economic and technological driving forces and how green house gas emissions are likely to vary [60]. We modeled *Bd* using the A2a storyline, a high emissions scenario, which describes a very heterogeneous world that is self-reliant, with a high rate of population growth, slow economic development that is regionally oriented and slow technological change compared to other storylines [61]. It also seems to be the storyline that is currently tracking actual climate changes.

## Results

### PCR testing for *Bd*

A total of 1018 skin swab samples were collected from amphibians in 39 sites located within 10 current or proposed protected areas in the Albertine Rift. A map of all the sites from which samples have been collected is shown in Fig 1. The prevalence of *Bd* in each of the current or proposed protected areas is listed in Table 1. Overall, 19.5% (199/1018) of the samples were PCR positive for *Bd*.

**Table 1:** *Bd* prevalence in sampled amphibians in current or proposed protected areas throughout the Albertine Rift.

Survey site	Country	Observed % Prevalence (95% CI, n)	Number Samples Tested
Budongo Forest	Uganda	15 (5.2–36.0, n = 3)	20
Bwindi Impenetrable Forest National Park	Uganda	18.8 (13.9–24.8, n = 37)	197
Nyungwe Forest National Park	Rwanda	16.6 (12.1–22.4, n = 33)	199
Cyamudongo, geographically isolated from, but part of Nyungwe Forest National Park	Rwanda	21.7 (9.7–41.9, n = 5)	23
Proposed Itombwe Forest Reserve and Balala Forest	DRC	24.2 (19.4–29.6, n = 64)	265
Kahuzi-Biega National Park (highlands)	DRC	58.5 (45.1–70.7, n = 31)	53
Kahuzi-Biega National Park (lowlands)	DRC	9.9 (5.9–16.2, n = 13)	131
Kisimba-Ikobo Reserve—West of Lake Edward	DRC	0 (0–32.4, n = 0)	8
Reserve des Gorilles de Punia: West of Kahuzi-Biega	DRC	21.4 (7.6–47.6, n = 3)	14
Luama Katanga Reserve and proposed Ngamikka Park (Misotshi-Kobogo massif, also known as Kobobo Plateau)	DRC	9.3 (5.1–16.2, n = 10)	108
Total for all areas		19.5 (17.2–22.1, n = 199)	1018

Of the 17 genera (*Afrixalus*, *Amietia*, *Amietophrynus*, *Arthroleptis*, *Boulengerula*, *Cardioglossa*, *Chiromantis*, *Hemisus*, *Hylarana*, *Hoplobatrachus*, *Hyperolius*, *Kassina*, *Leptopelis*, *Phlyctimantis*, *Phrynobatrachus*, *Ptychadena*, *and Xenopus*) that were sampled for the presence of chytrid fungus in the Albertine Rift, 10 genera had samples that were *Bd* positive and 7 genera were negative (*Boulengerula*, *Cardioglossa*, *Chiromantis*, *Hemisus*, *Hylarana*, *Hoplobatrachus*, and *Kassina*). Very few samples were collected from *Bd*-negative genera (n = 25 swabs for 7 genera combined). Shown in Table 2 is the prevalence for each genus that was positive for *Bd*. Amphibians from the genus *Afrixalus* and *Leptopelis* had the highest prevalence of *Bd* (29.9 and 29.4% respectively).

**Table 2:** Prevalence of *Bd* positive genera in tested samples (CI, Wilsons score).

Genera	Number Sampled	Positive	Negative	Observed % Prevalence (95% CI)
*Afrixalus*	87	26	61	29.9 (21.3–40.2)
*Amietia*	45	10	35	22.2 (12.5–36.3)
*Amietophrynus*	30	1	29	3.3 (0.6–16.7)
*Arthroleptis*	86	7	79	8.1 (4.0–15.9)
*Hyperolius*	433	89	344	20.6 (17.0–24.6)
*Leptopelis*	126	37	89	29.4 (22.1–37.8)
*Phlyctimantis*	5	1	4	20.0 (3.6–62.4)
*Phrynobatrachus*	82	13	69	15.9 (9.5–25.3)
*Ptychadena*	53	8	45	15.1 (7.1–27.1)
*Xenopus*	46	7	39	15.2 (7.6–28.2)

### Analysis of the ITS1-5.8S-ITS2 region of *Bd*

The ITS1-5.8S of *Bd* is a multi-copy region of the *Bd* genome and recent work has shown that copy number varies considerably (range of 10–144 copies with an average of 77 copies per zoospore) between different *Bd* strains [62]. Shown in S1 Fig are the number of copies/swab calculated for all the positive samples. Overall, 63.3% (126/199) of the *Bd* positive samples had less than 10,000 copies of the ITS1-5.8S region per swab, which would indicate low levels of zoospores (69–1000 zoospores/swab range) assuming the above estimated (10–144) copies per zoospore [62]. We found that 24.1% of the *Bd* positive samples (48/199) had 10,000–100,000 copies, and 8.5% (17/199) had 100,000–1,000,000 copies. We found that 4.0% (8/199) of the positive samples had very high ITS1-5.8S copy numbers in the range of 1,000,000–10,000,000. Overall, 82.9% (165/199) of the positive samples had less than 50,000 copies/swab, which, depending on the types of *Bd* strain(s) in each sample, would place these zoospore loads below the range of 347–5000 zoospores/swab assuming the above estimated range of copies per zoospore [62].

**Fig 2:**
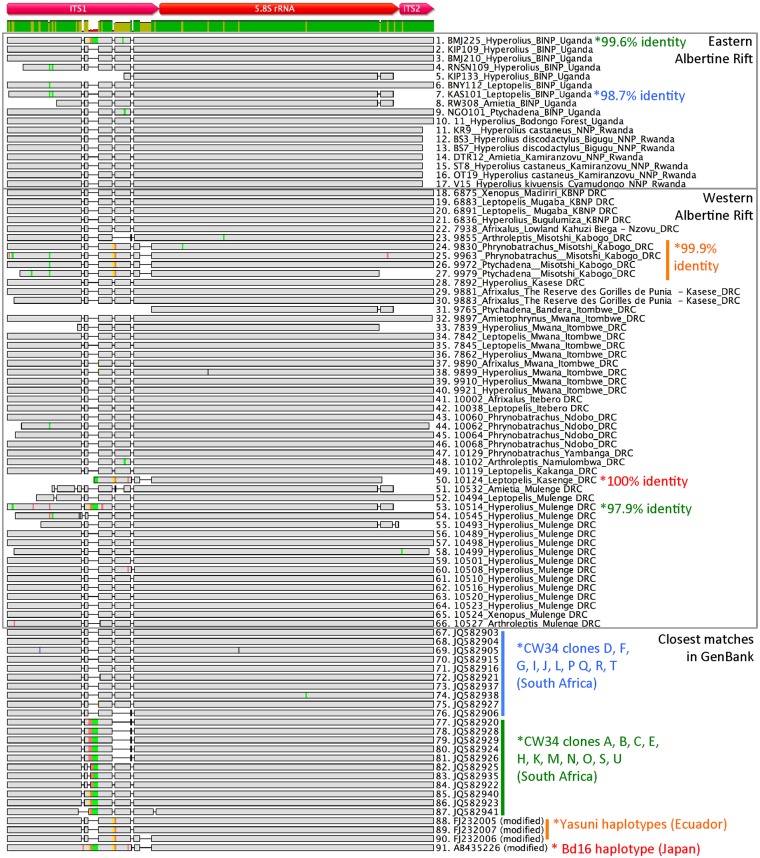
DNA sequence alignment of the ITS1-5.8S-ITS2 region of *Bd*. The color labels on the bottom right indicate the *Bd* strain and haplotypes found in GenBank, and sequences marked with an asterick with matching color indicates the percent identity of that sequence to the corresponding GenBank haplotype. Colors within the sequences denote differences in base pairs between sequences in the alignment (Adenine = red, Thymine = green, Guanine = yellow and Cytosine = blue). Grey regions indicate nucleotides that are identical in the alignment. Sample identification and GenBank accession numbers are shown.

Samples positive for *Bd* using qPCR were retested by conventional PCR using primers that amplify a 300 bp region spanning the ITS1-5.8S-ITS2 region of *Bd* [50]. A subset of positive PCR products were directly sequenced and analyzed, and compared to each other and to known *Bd* sequences. Shown in Fig 2 is a DNA alignment of the ITS1-5.8S-ITS2 region of *Bd* from 66 *Bd*-positive samples separated by region (western and eastern Albertine Rift) and their matching sequences obtained from GenBank. Overall, 89.3% (59/66) of the sample sequences from both the western and eastern Albertine Rift were >99% identical to a Global Panzootic Lineage (Bd-GPL-2) strain CW34 isolated from a *Xenopus* sp. from South Africa, indicating very little variation in *Bd* haplotype diversity in this area of the *Bd* genome between the eastern and western side of the Albertine Rift region, and the region as a whole. We found that 87.7% (58/66) of the Albertine Rift sequences were >99% identical to CW34 group 1 (clones D, F, G, I, J, L, P Q, R and T) found in GenBank, and 1.5% (1/66) of the sequences were >99% identical to the CW34 group 2 (clones A, B, C, E, H, K, M, N, O, S and U). Only 3% (2/66) of the sequences were between 97.9% and 98.7% identical to CW34, which was the closest match found in GenBank. Although the majority of sequences closely matched the South African *Bd* CW34 strain, we also found sequences that did not match, and were more closely related to strains found in other parts of the world. We found that 6% (4/66) of Albertine Rift *Bd* sequences were >99% identical to a strain of *Bd* found in Yasuni National Park in Ecuador, and all of these sequences were recovered from *Phrynobatrachus* and *Ptychadena* sp. frogs collected in Misotshi-Kobogo, DRC. One sequence recovered from a *Leptopelis* sp. in North Balala, DRC, was 100% identical to the Bd16 strain found in a *Ceratophrys cranwelli*, a South American species of frog that was that was bred in Japan from imported frogs [63].

### Assessing amphibians for the disease chytridiomycosis

**Fig 3:**
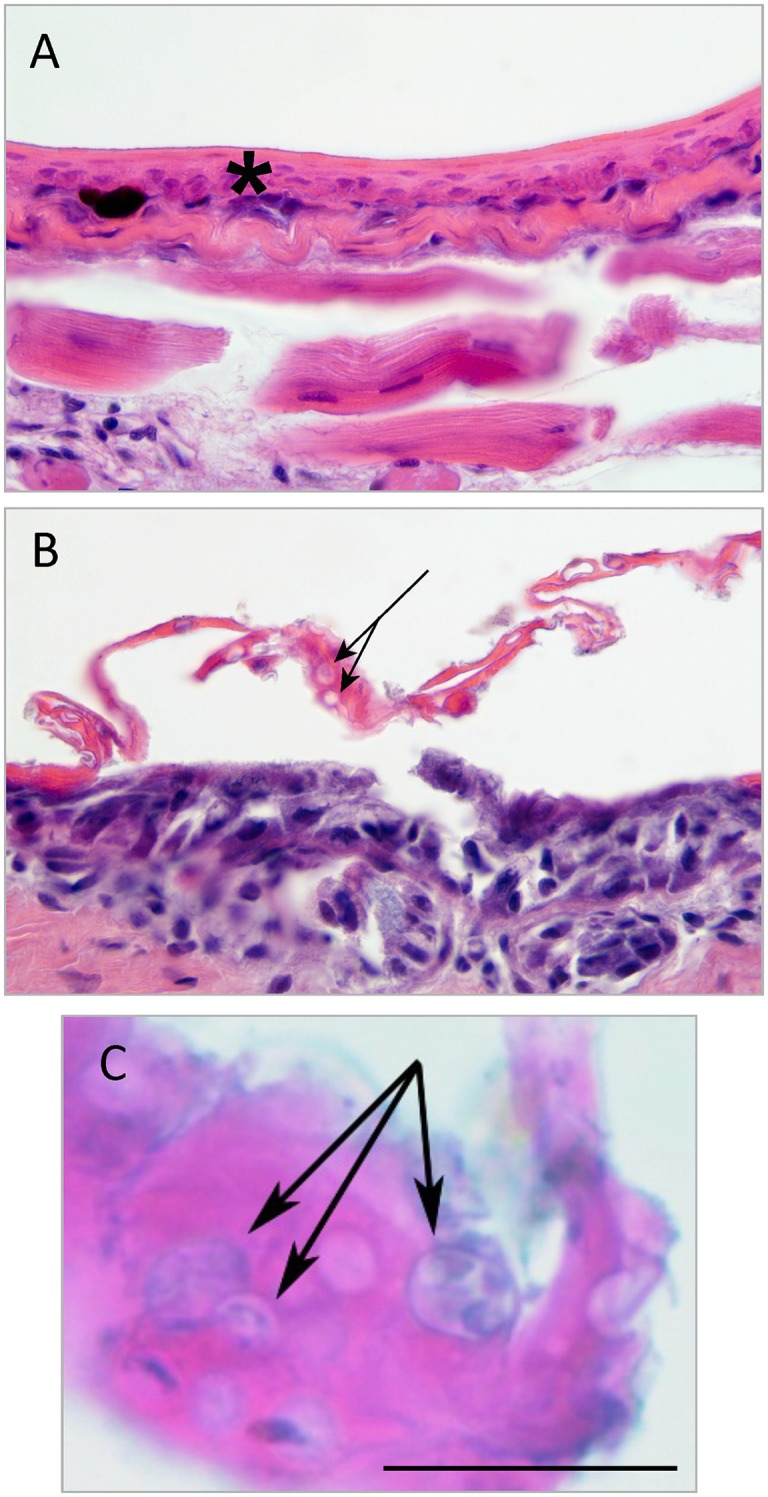
Skin histology. A–C. Skin section from the ventral hind limb skin of *Ptychadena sp*. 9977 (A) and 9979 (B, C). A. Normal skin, including thin epidermis (asterisk) and underlying skeletal muscle (1000X). B–C. Abnormal skin. Histological findings include moderate epidermal thickening and cellular disorganization (hyperplasia), mild hyperkeratosis with moderate numbers of intracorneal empty or zoospore-containing chytrid thalli (B, C; arrows; scale bar = 20μm).

Histologic examination of skin samples was performed to identify *Bd* and chytridiomycosis. Of the 163 skin samples obtained from individual specimens, two (No. 9979, *Ptychadena sp*. from Ngamikka NP (Misotshi-Kobogo) and No. 7892, *Hyperolius sp*. from the lowlands of Kahuzi-Biega National Park, in DRC) had epidermal changes consistent with chytridiomycosis, including mild to moderate epidermal hyperplasia and hyperkeratosis, and numerous intracorneal zoospore-containing or empty chytrid thalli consistent with *Bd* and suggestive of chytridiomycosis (Fig 3).

Both animals were also PCR-positive for *Bd*; sample No. 9979 (*Ptychadena*) had 2843 copies (514 STDEV) and sample No. 7892 (*Hyperolius*) had 3,131,439 (1,228,693 STDEV) copies of the ITS1-5.8S region per swab (S1 Fig, red bars). Shown in Table 3 are the results for all the PCR-positive animals that were also assessed by histology.

**Table 3:** Genera assessed for the disease chytridiomycosis that were also *Bd* positive by PCR.

Genus and Sample (Sample IDs)	Number of *Bd* PCR Positive Samples (copy number range/swab)	Number Positive for Chytridiomycosis (by histology)	% Positive for Chytridiomycosis
***Afrixalus* sp. (9734, 9774, 9782, 9883)**	**4 (308–195,005)**	**0**	**-**
***Hyperolius* sp. (7862, 7892, 9742, 9752, 9784, 9790, 9792, 9847, 9973)**	**9 (144–3,131,439)**	**1**	**11.1**
***Leptopelis* sp. (9770)**	**1 (190)**	**0**	**-**
***Phlyctimantis* sp. (9738)**	**1 (186)**	**0**	**-**
***Phrynobatrachus* sp. (MUSE 10068, 9830)**	**2 (7580–294,562)**	**0**	**-**
***Ptychadena* sp.(9732, 9979, 9765, 9733)**	**4 (144–119,378)**	**1**	**25.0**
	**21**	**2**	**9.5**

A full necropsy was performed on one amphibian that was found dead during our surveys (MUSE10068). This animal, a 6.07g adult *Phrynobatrachus* sp. (puddle frog), was collected February 25th, 2014 from the Balala Forest in eastern DRC at 2171 m in elevation. A skin swab from this animal was positive for *Bd*. Histologic evidence of chytridiomycosis was not seen in skin from multiple areas including the feet. Pathologic processes to explain the death of the frog were not identified in any of the examined tissues. No zoospores were identified in examined pieces of skin, and the calculated copies of the ITS1-5.8S region per swab was 294,562 (21,613 STDEV). These combined results suggest a subclinical infection and/or possible carrier state.

### Assessing historical presence of *Bd*

We analysed 209 voucher specimens collected between 1925–1951 to investigate the historical presence of *Bd* in the western Albertine Rift. Overall 88% (184/209) of the samples had amplifiable 16S rDNA, and 25 samples were deemed indeterminate because amplification failed. Of 68 samples collected between 1925–1934, 67 were successfully amplified for 16S rDNA (98.5% success in DNA amplification). Of 141 samples collected between 1949–1951, 117 were successfully amplified for 16S (83.0% success in DNA amplification). Interestingly, we found higher success in rDNA amplification in the older samples. Results are shown in S1 Table. Of the 184 16S-positive samples, only 1 (AM130; ID108878) was positive for *Bd*. DNA sequencing and BLAST analysis of this 97bp fragment was 98% identical to *Bd* strain CW34 (Clones G, I, J, P and T). Attempts to amplify larger segments of the ITS1-5.8S-ITS2 region were unsuccessful. The positive sample was collected from an Itombwe river frog (*Phrynobatrachus asper*) collected by R. Laurent in August 1950, near the Makenda River in the Itombwe highlands. This finding represents the oldest record of *Bd* in the DRC, and it confirms its presence in this region as early as 1950.

Skin swab samples were also collected from 49 amphibians collected between 1965–2013 that were archived at Makerere University. Overall, amphibian mitochondrial 16S rDNA was amplified in 78% (38/49) of these samples; 11 samples had indeterminate results due to failure to amplify the 16S gene fragment. Of the 16S-positive samples, 7.9% (3/38) were positive for *Bd*. All positive samples were from amphibians collected in the Budongo Forest in 2013 (S2 Table).

### Modeling the present and future distribution of *Bd* in the Albertine Rift

**Fig 4:**
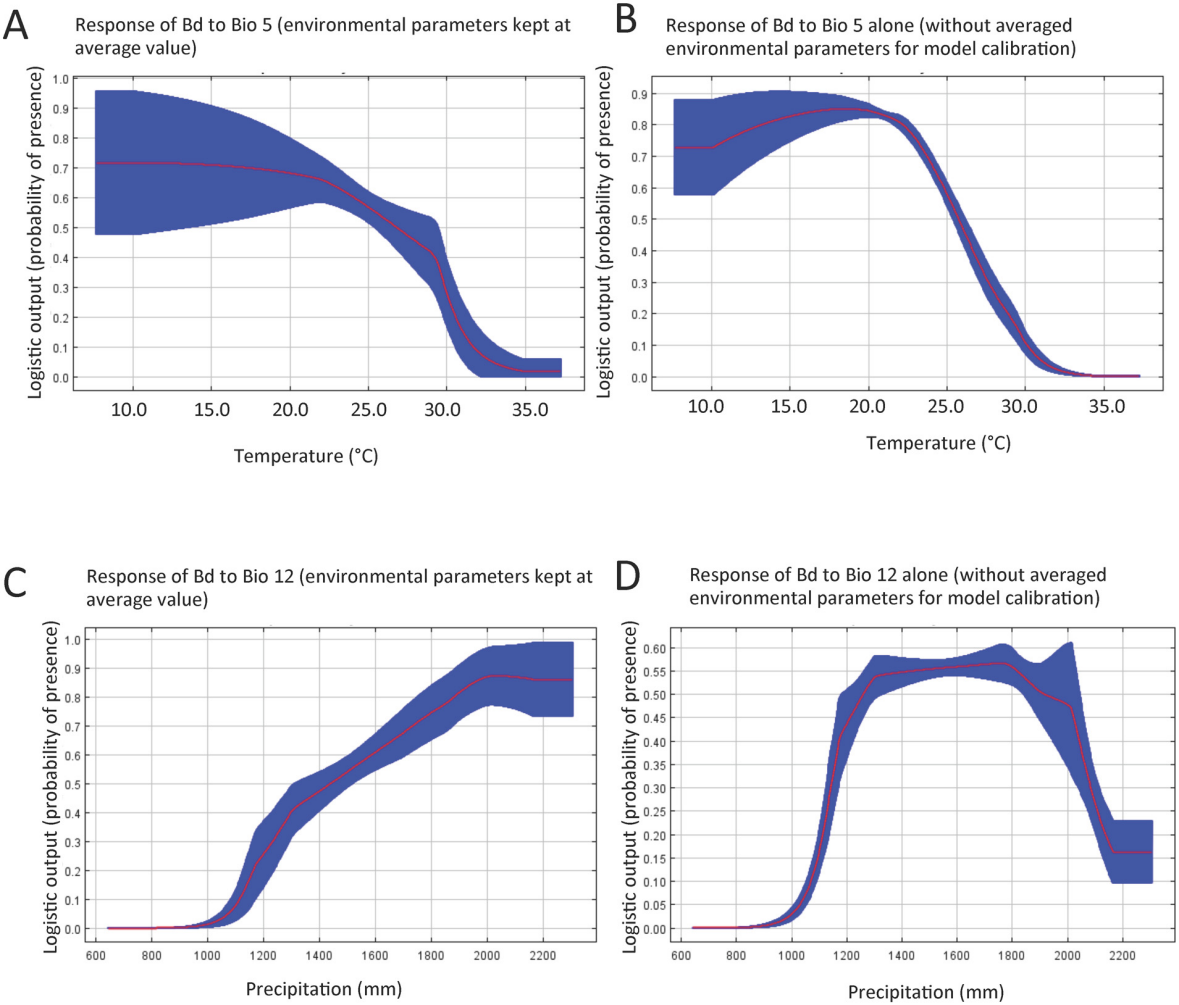
MaxEnt response curves illustrate how the likelihood for *Bd* occurrence is influenced by maximum temperature of warmest month (Bio5) and the mean annual precipitation (Bio12). A. Shows the probability of presence of *Bd* as it relates to Bio5 when all other environmental variables are kept at their average values. B. Shows the same as in A, except that Bio5 alone was used for model calibration. C. Shows the probability of presence of *Bd* as it relates to Bio12 when all other environmental variables are kept at their average values. D. Shows the same except that Bio12 alone was used for model calibration. The red line is the mean of 100 bootstrap runs with +/- one standard deviation (blue).

Species distribution models (SDM’s) have been used to predict the potential distribution of *Bd* over large regions [9,23,26–28,64,65]. We used our PCR data gathered from herpetological surveys conducted between 2010–2015 and gridded WorldClim interpolated climate surfaces to model which parameters exert the greatest control over *Bd* distribution. The host-pathogen interaction of *Bd* has a high degree of climatic sensitivity, particularly to temperature [58,66–71]. The results of the MaxEnt modeling suggest that *Bd* distribution is mostly affected by temperature and rainfall, which together account for 70% of the model. Variable contributions for each predictor after 100 bootstrap runs were as follows: Bio5 (maximum temperature of the warmest month), 52.5%; Bio12 (mean annual precipitation), 17.5%; Bio2 (mean diurnal range), 11.1%; Bio17 (precipitation of driest quarter), 9.8%; Bio6 (min temperature of coldest month), 3.3%; Bio16 (precipitation of wettest quarter), 3.2%; and Bio7 (temperature annual range), 2.5%. We found that Bio5 produces the greatest contribution to model performance when used in isolation, which was similar to results obtained with Bio5 when the other environmental parameters were kept at average value (Fig 4A and 4B). This result shows that Bio5 contains information by itself that is critical in defining the distribution of *Bd* occurrence in the Albertine Rift. Bio12 reduces model performance the most if omitted, indicating that it has infomation not present in the other variables used in the model. Based on our model predictions, the optimal range of maximum temperature of the warmest month for the probability of presence of *Bd* is between 10–21°C, and the probability of occurrence decreases rapidly above 22°C (Fig 4A and 4B). The MaxEnt results also indicate that the probability of *Bd* presence in the study area increases with higher mean annual precipitation when all other environmental parameters are kept at the average value (Fig 4C). When Bio12 is used for model calibration without other environmental parameters, we found that likelihood of *Bd* presence decreases sharply when annual rainfall exceeds 1800mm (Fig 4D). Based on our model, optimal annual precipitation for the habitat suitability of *Bd* ranges between 1300mm to 1800mm. (Fig 4D).

In our initial MaxEnt SDM run, we used presence-only datapoints from a total of 634 individuals that were tested for *Bd*. Samples were collected from Kahuzi-Biega (n = 53), Itombwe (n = 54), Ngamikka (n = 108), Nyungwe (n = 222) and Bwindi (n = 197). The scale of the modeling was set at 1 km^2^, which meant that some presence records were found in the same cells as others. Therefore, a total of 40 chytrid presence records were used in the MaxEnt model fitting. The results of the outputs are given in S2A Fig and show the predicted extent of *Bd* using our original data.

We then tested the model on samples from areas that had not been previously surveyed, focusing on the northern part of the Albertine Rift and the lowlands. We used as inputs to test our model samples from six field expeditions were that were conducted in 2013 where 384 individuals were tested for *Bd*: Itombwe and North Balala Forest (n = 211), Kisimba-Ikobo Community Reserve (n = 8), Punia Gorilla Reserve (Kasese-west of Kahuzi-Biega National Park, n = 14), lowland Kahuzi-Biega National Park (Nzovu, Kasese, Itebero n = 131), and Budongo Forest (n = 20). Chytrid presence locations from these samples were combined with locations provided by E. Greenbaum (S4 Table [30–32]) and from Uganda (Bd-maps.net, [29]), then analyzed through MaxEnt modeling. A total of 48 chytrid presence records were used for testing the previous model. The model results show that our original data correctly predicted 34 out of the 48 (70.8%) new locations, but did not successfully predict areas in Kahuzi-Biega lowland sector and also in the Katanga province in the southern Albertine Rift near the Zambian border (S2A Fig). However, by incorporating this new set of observations from a more diverse set of localities where *Bd* presence has been confirmed we have rerun and trained this model, and thus the range of predicted *Bd* distribution has expanded (S2B Fig). The *Bd*-positive datapoints used in the predictive modeling are shown in S3 and S4 Tables. Some caution should be used with interpretation of these results as very few data points were collected from lower elevations. Further work is therefore needed to assess the prevalence of this disease in tropical Africa at elevations below 600m ASL.

**Fig 5:**
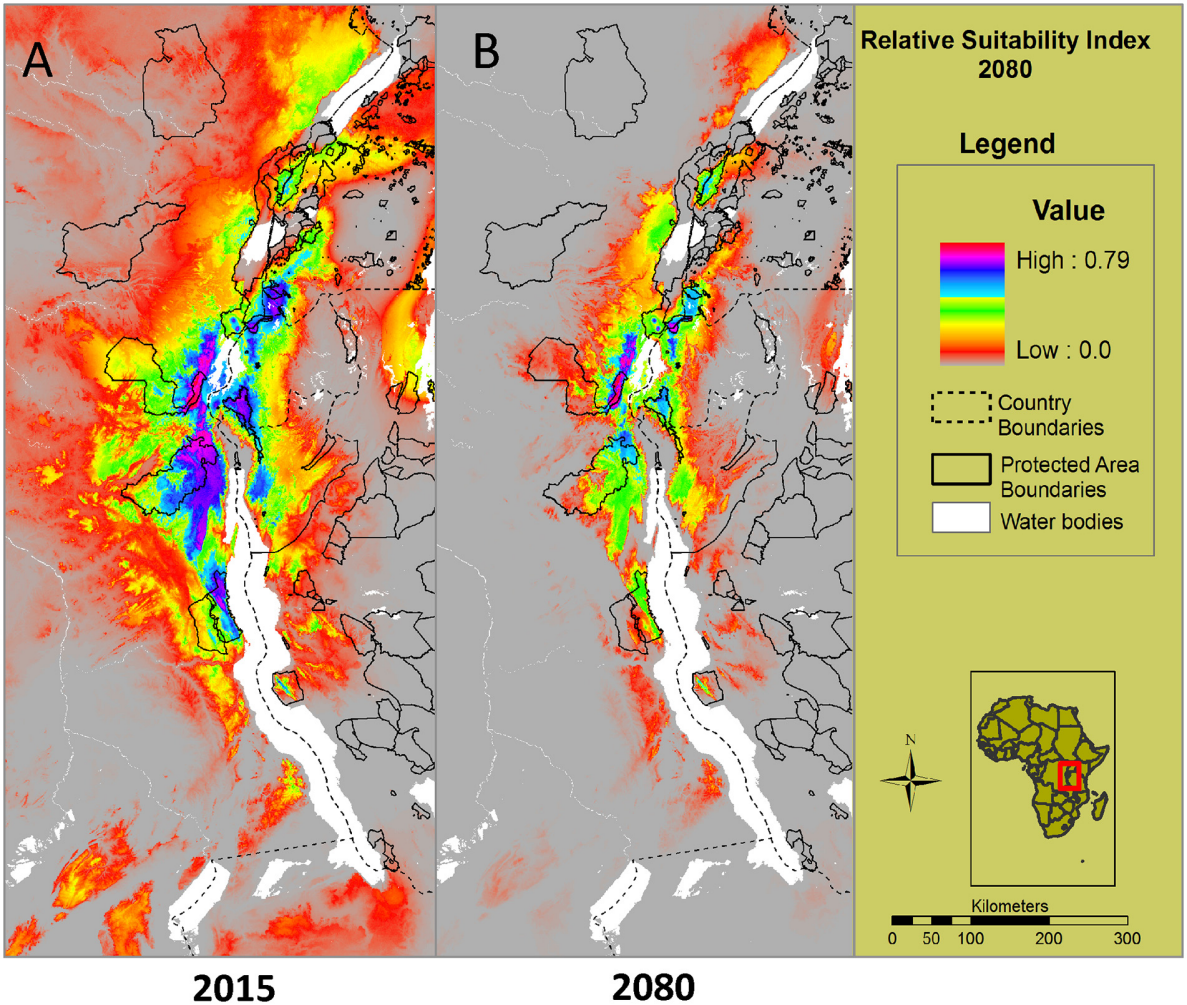
Predicted future habitat suitability and *Bd* distribution in 2080. A. Illustrates the predicted current distribution and risk of *Bd* to amphibians in 2015 using all locations in the modeling (*Bd* records obtained in this study and previous studies [29–32]). B. An average of the model output from the three General Circulation Models that shows the future distribution of where amphibians are likely to be at risk for *Bd* infection in 2080 under the A2a scenario. Our results predict a large range contraction of suitable habitat for *Bd* with future climate change.

These results were then applied to the future climate states of the A2a scenario for the year 2080. Under these conditions, MaxEnt predicts a major range contraction of suitable habitat for *Bd* by 2080 with high to moderate habitat suitability only occurring in the higher elevations both east and west of the Rift (Fig 5). This accompanies a major contraction of suitable habitat for amphibians in the Albertine Rift (Plumptre *et al*. in prep) potentially exacerbating large declines in the populations of the species confined to the highland areas.

## Discussion

*Bd* is listed by the World Organization for Animal Health (OIE) as a reportable disease [72] and is considered a significant conservation threat in many amphibian species around the world. Our results identifying *Bd* in and across the Albertine Rift are consistent with results found by others, and also expand the known *Bd* distribution in the Albertine Rift [29–32]. In our study, 19.5% of the 1018 amphibians from the Albertine Rift sampled between 2010–2014 tested positive for *Bd*. This is somewhat lower than previous observations, which found an overall prevalence of 34.9% in the eastern DRC samples collected between 2008–2011 [31]. Across other regions in Africa, researchers have found a 31.5% prevalence in Kenya [20], 19–36% in Gabon [24], and 14.8% in South Africa [23]. Most of these studies, where zoospore genomic equivalents were measured, showed low to moderate zoospore loads (<1375 GE); and evidence to assess the disease chytridiomycosis by histopathology in wild African species is limited [28,30,73]. Histopathology is a critical addition in any retrospective or prospective study to confirm the presence of chytridiomycosis, as *Bd* infection alone does not always result in disease or mortality, and because identification of clinically healthy carriers through histology can inform recovery, reintroduction, and translocation of individuals or groups of animals in conservation projects.

Several species, such as the bullfrog (*Lithobates catesbeianus*) and African clawed frog (*Xenopus laevis)* have been shown to have low susceptibility to the disease chytridiomycosis when infected with *Bd*. These, and other low susceptibility species, may act as carriers and be a source of exposure to naïve and/or highly susceptible species [74–77]. Gross examination and histopathology of skin samples from voucher specimens combined with the low to moderate *Bd* levels in the majority of the positive samples tested using PCR (63.8% of all positive samples had less than 10,000 copies of the ITS1-5.8S region per swab), suggest that, with the exception of specimens 9979 (*Ptychadena*) and 7892 (*Hyperolius*), the majority of the animals that were *Bd* positive did not develop the disease chytridiomycosis despite being infected with *Bd*. In addition, over our four-year study, only one dead amphibian was encountered and none of the animals handled exhibited clinical signs suggestive of chytridiomycosis (lethargy, lack of righting reflex, excessive shedding of skin). It is therefore our presumption that no significant mortality events have occurred in this study area during the current study period. However, confidence in this conclusion is limited by the relatively small sample size relative to the geographic region of study and lack of continuous monitoring and testing. Mortality events may also be cryptic depending on the species and geographic location, and how quickly the carcasses decay before the event is noticed. In addition, this conclusion cannot be applied to amphibians in areas outside of the study sites or species. Based on the current state of our knowledge and when taken together with other studies looking at species within the same genera across Africa (*Afrixalus*, *Arthroleptis*, *Hyperolius*, *Leptopelis*, *Phlyctimantis*, *Ptychadena*, and *Phrynobatrachus*) [19,20,23,24,27], there is no current evidence to suggest *Bd* infection or chytridiomycosis is causing large die-offs or significant population effects across the Albertine Rift [29–32]. Our field surveys and these other studies however, provide only a snapshot of *Bd*-prevalence at a certain point in time and time of year, and do not rule out the possibility of *Bd*-induced mortality as a possible contributing significant factor in animal health or as a factor that could negatively affect population dynamics in the past or future. Many factors, including introductions of new strains of *Bd* or pathogen mutation towards increased virulence, could result in mortality events within or across species, or situations in which sub-clinical infections may cross a threshold for terminal disease [78–81]. Our data provide important baseline information for comparative studies should any mortality events be encountered in the future.

The earliest records of *Bd* in historical specimens are from Brazil in 1894 [82], Illinois in the United States in 1888 [83], and from Africa in *Xenopus laevis* collected in Cameroon in 1933, Uganda in 1934, and South Africa in 1938 [33,74,84], and our new finding of *Bd* in an Itombwe river frog (*Phrynobatrachus asper*) from the DRC in 1950. The Itombwe river frog is currently listed as data-deficient by the IUCN and was thought to have vanished until it was rediscovered in the Itombwe highlands in 2009. When rediscovered, one of two live *P*.*asper* tested was positive for *Bd* [31,85]. These results suggest that *Bd* has existed in the Albertine Rift for at least 65–80 years in DRC and Uganda respectively [33].

DNA sequence analysis of the ITS1-5.8S-ITS2 region of *Bd* shows that the Albertine Rift strains closely match *Bd* sequences from South Africa (CW34), Ecuador (Yasuni) and Japan (Bd-16). Given the wide, intercontinental distribution of these strains, questions remain as about their origin. ITS1-5.8S-ITS2 is a multicopy region, and many haplotypes have been found to exist within *Bd* strains such as the CW34 strain [86]. Our PCR products were not cloned and sequenced, and we therefore cannot speculate on how many haplotypes may exist within individual strains of *Bd* found in the Albertine Rift. However, our results suggest that sequences we have identified are closely related to haplotypes previously recovered from the CW34 *Bd* strain and that the vast majority match one particular group of clones. CW34 is thought to have originated in Namaqualand, South Africa and was isolated from a *Xenopus laevis* in 2005 [86]. The lineage groups phylogenetically within the globally dispersed clade called *Bd*-GPL-2 (Global Panzootic Lineage)[86], and this cluster contains the most geographically diverse and genetically similar group of known *Bd* strains. *Bd*-GPL-2 is found in Africa, Australia, Asia, and North and Central America [79,86,87]. Molecular clock analysis places an estimate of the emergence of the Global Panzootic Lineage strains at 10,000–40,000 years ago [79].

Our molecular diagnostic results coupled with collection of amphibians that appeared on examination to be in good health, absence of apparent die-offs or many dead animals, and lack of chytridiomycosis as a common histologic finding over the several years of our surveys, are consistent with what others have suggested about *Bd* as being enzootic and likely native to Africa. Future investigations into potential strain variations of *Bd* in these species and their populations through genomic analysis will be critical to understand if co-evolutionary relationships exist with the Albertine Rift *Bd* strains with their respective host genera, and may provide new insight into the complex and controversial origin of this organism, and the emergence and spread of it’s various lineages [43].

Earlier work by other research groups have used a MaxEnt approach to model the habitat suitability of *Bd* across large areas, and predicted that *Bd* should be widespread in the Albertine Rift region [11,27,28]. However, at the time these studies were conducted very little testing in the region was available to validate these models. Our results provide valuable data points that increase the resolution of this type of modeling, and by sampling new areas allowed us to test the certainty and train the model for improved predictability. However, with any model come certain caveats. WorldClim variables are based on interpolation of long-term climatological records onto a high-resolution (1 km) grid. Such records are especially sparse across the Albertine Rift, which makes accurate representation of WorldClim climatic variables away from observing sites questionable. This is more a concern for precipitation and other moisture variables than it is for temperature. The latitudinal belt of the Albertine Rift falls within the inner tropics, which means annual temperature is strongly influenced by surface elevation, and to lesser degrees by proximity to major water bodies and land surface type. Thermal conditions in WorldClim reflect grid point elevations, which are quite accurate, so the WorldClim temperature fields in the Rift corridor likely represent a fair estimation of reality. The Bio5 variable, maximum temperature of the warmest month, explains 52.5% of the variance in *Bd* occurance, and is unsurprising given the recognized thermal constraints over *Bd* distribution.

Another limitation of the MaxEnt approach is the use of annual precipitation sum to characterize rainfall rather than variables that represent seasonality. Mean annual precipitation (Bio12) was the second variable that highly influenced our model (17.5% of the explanatory power). The timing and duration of wet and dry seasons are heterogeneous across the Albertine Rift [40,88]. It is unknown how infection rates or pathogen loads might similarly vary with precipitation seasonality across the Albertine Rift because our sites were visited once, and whether such a factor might have influenced the results from the various field sampling initiatives. For example, recent studies found that amphibian *Bd* infection loads increase during dry seasons, likely due to the densification of populations and reduced water flow when ephemeral water bodies disappear with seasonal desiccation [38,89]. Our surveys also focused on collecting adult and juvenile amphibians, and we did not test for *Bd* in mouthparts of tadpoles. Tadpoles have been shown to act as a host for maintaining *Bd* in the environment [38]. If such information could be ascertained, it should become possible to develop more nuanced predictive models that also incorporate factors like amphibian distributions, whether or not tadpoles could be maintaining *Bd* in the environment, reproductive aggregations and behavior, aquatic parameters such as proximity to drainage, and vegetation parameters in the wet and dry seasons.

Our results using MaxEnt modeling indicate that the highest habitat suitability for *Bd* is found in the highlands of the Albertine Rift, with moderate to low habitat suitability extending into the lowlands. Although our modeling results are also consistent with the known environmental preferences of *Bd* relative to temperature and rainfall based on previous studies [9,35,38,59,67,90], the results we obtained from testing and training this model indicates that more work needs to be done in the lowlands of the Congo Basin (<600m) to better refine our model and understand the lower elevation and higher temperature limits on *Bd* infectivity in its natural environment.

Climatic model predictions indicate significant temperature and precipitation increases across the Albertine Rift in the 21st century [40–42]. An 11-member multi-model ensemble from GCMs used in the IPCC Fourth Assessment Report, downscaled to 50 km spatial resolution, demonstrates relatively monotonic thermal increases across the Albertine Rift throughout the 21st century under the moderate B1 and more severe A2 global emissions scenarios, with little seasonal or spatial variation evident [40–42]. In contrast, precipitation changes are much more complex. Averaged across the domain, mean annual precipitation is projected to increase by 17.26% (1199 mm vs. 1406 mm) by 2090 relative to the baseline year 1990 [40]. These increases are not distributed evenly throughout the year. Most notably, in the southern sections of the Rift large precipitation increases in the November–January months shorten the duration of the dry season. Under our current model, the likelihood of *Bd* occurrence is predicted to decrease during warmer periods, and when precipitation exceeds an annual rainfall threshold above 1800mm per year, so we infer that *Bd* prevalence may decrease as a result of climate change. This is borne out by the MaxEnt modeling we have presented here, which indicates a major range contraction of habitat suitability for this fungus by the end of the century.

Our database and sample archive can now be used as a reference when monitoring for new strains of *Bd* over time, and to determine if the occurrence of this pathogen changes seasonally or under wetter and warmer climatic conditions. More fundamentally, we still need to determine if amphibians from the Albertine Rift possess innate resistance or tolerance to *Bd*. If these amphibian species are indeed naturally immune to disease from *Bd* infection, there is much to be learned from exploratory research focused on amphibian metagenomics, understanding the skin microbiome diversity, and the innate immunity of African species. The Albertine Rift is one of the world’s hotspots for amphibian biodiversity, and is also one of the most threatened. Baseline data on *Bd* can help to form a more complete picture of the presence and significance of this fungus and help guide and inform discussions on climate and species-related conservation strategies at both the local and global levels.

## Supporting Information

S1 Dataset FASTA file. DNA sequence alignment of partial ITS1-5.8S-ITS2 sequences used in Fig 2.
(FASTA)

S1 Fig Calculated copy numbers in positive samples. Log-scale graph showing all *Bd*-positive samples plotted against the calculated number of copies of the ITS1-5.8S region per swab. Error bars indicate the standard deviation (positive only) for each triplicate sample. Red bars indicate which two samples had corresponding skin tissue analyzed and had histological changes consistant with the disease chytridiomycosis.
(TIFF)

S2 Fig Trained model of the current relative habitat suitability and distribution of *Bd*. A. (Initial model). Current distribution of areas where amphibians are likely to be at risk for *Bd* infection using original records (maroon hexagons). All areas predicted as suitable where the new positive localities (green hexagons) appear indicate areas where we had not sampled but the model predicted as a potential suitable habitat. These areas include (Budongo, Kibale, Kamengo, Lake Bunyonyi, North Balala Forest, parts of Itombwe Massif). The model didn’t predict the lowlands of Kahuzi-Biega National Park and Katanga province. B. An updated model showing the current distribution of areas where amphibians are likely to be at risk for *Bd* infection when using both original (maroon hexagons) and new occurrence (green hexagons) records for training.
(TIFF)

S1 Table Summary of *Bd* results from historical specimens. Elevations are in meters above sea level.
(DOCX)

S2 Table PCR results of historical samples collected from Makerere University in Uganda. Elevations are in meters above sea level.
(DOCX)

S3 Table Sample ID, Genus, Date of collection, and GPS location of all *Bd* positive samples included in the modelling analysis. Elevations are in meters above sea level.
(DOCX)

S4 Table Additional *Bd*-positive localities provided by Bd-maps.net and E. Greenbaum that went into the modelling analysis [29–32,49]. Elevations are in meters above sea level.
(DOCX)

## References

[1] Brooks T, Balmford A, Burgess N, Fjeldsa J, Hansen LA, Moore J, et al Towards a blueprint for conservation in Africa. Bioscience. 2001;51: 613–624.

[2] Plumptre AJ, Davenport TRB, Behangana M, Kityo R, Eilu G, Ssegawa P, et al The Biodiversity of the Albertine Rift. Biol Conserv. 2007;134: 178–194.

[3] Portillo F, Greenbaum E. At the edge of a species boundary: A new and relatively young species of Leptopelis (Anura: Arthroleptidae) from the Itombwe Plateau, Democratic Republic of the Congo. Herpetologica. 2014;70: 100–119.

[4] Portillo F, Greenbaum E. A new species of the Leptopelis modestus complex (Anura: Arthroleptidae) from the Albertine Rift of central Africa. J Herpetol. 2014;48: 394–406.

[5] Evans B, Carter T, Tobias M, Kelley D, Hanner R, Tinsley R. A new species of clawed frog (genus Xenopus) from the Itombwe Massif, Democratic Republic of the Congo: Implications for DNA barcodes and biodiversity conservation. Zootaxa. 2008;1780: 55–68.

[6] Evans BJ, Greenbaum E, Kusamba C, Carter TF, Tobias ML, Mendel SA, et al Description of a new octoploid frog species (Anura: Pipidae: Xenopus) from the Democratic Republic of the Congo, with a discussion of the biogeography of African clawed frogs in the Albertine Rift. J Zool. 2011;283: 276–290. 10.1111/j.1469-7998.2010.00769.x PMC3086292 21546992

[7] Maximilian Dehling J. An African glass frog: A new Hyperolius species (Anura: Hyperoliidae) from Nyungwe National Park, southern Rwanda. Zootaxa. 2012; 53–64.

[8] Muths E, Pilliod DS, Livo LJ. Corrigendum to “Distribution and environmental limitations of an amphibian pathogen in the Rocky Mountains, USA” Biological Conservation. 2008; 141: 1484–1492. 10.1016/j.biocon.2008.09.012

[9] Puschendorf R, Carnaval AC, Vanderwal J, Zumbado-Ulate H, Chaves G, Bolaños F, et al Distribution models for the amphibian chytrid Batrachochytrium dendrobatidis in Costa Rica: Proposing climatic refuges as a conservation tool. Divers Distrib. 2009;15: 401–408.

[10] Rosenblum EB, Voyles J, Poorten TJ, Stajich JE. The deadly chytrid fungus: A story of an emerging pathogen. PLoS Pathog. 2010;6: 4–6. 10.1371/journal.ppat.1000550 PMC2813266 20126439

[11] Olson DH, Aanensen DM, Ronnenberg KL, Powell CI, Walker SF, Bielby J, et al Mapping the global emergence of Batrachochytrium dendrobatidis, the amphibian chytrid fungus. PLoS One. 2013;8 10.1371/journal.pone.0056802 PMC3584086 23463502

[12] Stuart SN, Chanson JS, Cox N a, Young BE, Rodrigues ASL, Fischman DL, et al Status and trends of amphibian declines and extinctions worldwide. Science. 2004;306: 1783–1786. 10.1126/science.1103538 15486254

[13] Voyles J, Young S, Berger L, Campbell C, Voyles WF, Dinudom A, et al Pathogenesis of chytridiomycosis, a cause of catastrophic amphibian declines. Science. 2009;326: 582–585. 10.1126/science.1176765 19900897

[14] Skerratt LF, Berger L, Speare R, Cashins S, McDonald KR, Phillott AD, et al Spread of chytridiomycosis has caused the rapid global decline and extinction of frogs. Ecohealth. 2007;4: 125–134. 10.1007/s10393-007-0093-5

[15] Fisher MC, Garner TWJ, Walker SF. Global emergence of *Batrachochytrium dendrobatidis* and amphibian chytridiomycosis in space, time, and host. Annu Rev Microbiol. 2009;63: 291–310. 10.1146/annurev.micro.091208.073435 19575560

[16] Gower DJ, Doherty-bone TM, Aberra RK, Mengistu A, Menegon M, Sá De R, et al High prevalence of the amphibian chytrid fungus (Batrachochytrium dendrobatidis) across multiple taxa and localities in the highlands of Ethiopia. Herpetological Journal. 2012; 22: 225–233.

[17] Channing A, Finlow-Bates KS, Haarklau SE, Hawkes PG. The biology and recent history of the Critically Endangered Kihansi Spray Toad Nectophrynoides Asperginis in Tanzania. J East African Nat Hist. 2006;95: 117–138. 10.2982/0012-8317(2006)95[117:TBARHO]2.0.CO;2

[18] Imasuen AA, Weldon C, Aisien MSO, Dupreez LH. Amphibian chytridiomycosis: first report in Nigeria from the skin slough of Chiromantis rufescens. Froglog. 2009;90: 6–8.

[19] Reeder NMM, Cheng TL, Vredenburg VT, Blackburn DC. Survey of the chytrid fungus Batrachochytrium dendrobatidis from montane and lowland frogs in eastern Nigeria. Herpetol Notes. 2011;4: 83–86.

[20] Kielgast J, Rödder D, Veith M, Lötters S. Widespread occurrence of the amphibian chytrid fungus in Kenya. Anim Conserv. 2010;13: 36–43. 10.1111/j.1469-1795.2009.00297.x

[21] Doherty-Bone TM, Gonwouo NL, Hirschfeld M, Ohst T, Weldon C, Perkins M, et al Batrachochytrium dendrobatidis in amphibians of Cameroon, including first records for caecilians. Dis Aquat Org. 2013; 102, 187–194. 10.3354/dao02557 23446968

[22] Baláž V, Kopecký O, Gvoždík V. Presence of the amphibian chytrid pathogen confirmed in Cameroon. Herpetol J. 2012;22: 191–194.

[23] Tarrant J, Cilliers D, du Preez LH, Weldon C. Spatial assessment of amphibian chytrid fungus (Batrachochytrium dendrobatidis) in South Africa confirms endemic and widespread infection. PLoS One. 2013;8 10.1371/journal.pone.0069591 PMC3718833 23894506

[24] Bell RC, Gata Garcia AV, Stuart BL, Zamudio KR. High prevalence of the amphibian chytrid pathogen in Gabon. Ecohealth. 2011;8: 116–120. 10.1007/s10393-010-0364-4 21210295

[25] Bletz M, Rosa G, Crottini A, Courtois E, Schmeller D, Rabibisoa N, et al Widespread presence of the pathogenic fungus Batrachochytrium dendrobatidis in wild amphibian communities in Madagascar. Nat Commun. 2015;5: 1–10. 10.1038/srep08633 PMC4341422 25719857

[26] Rödder D, Kielgast J, Bielby J, Schmidtlein S, Bosch J, Garner TWJ, et al Global amphibian extinction risk assessment for the panzootic chytrid fungus. Diversity. 2009;1: 52–66. 10.3390/d1010052

[27] Rödder D, Kielgast J, Lötters S. Future potential distribution of the emerging amphibian chytrid fungus under anthropogenic climate change. Dis Aquat Organ. 2010;92: 201–207. 10.3354/dao02197 21268982

[28] Penner J, Adum GB, McElroy MT, Doherty-Bone T, Hirschfeld M, Sandberger L, et al West Africa—A Safe Haven for Frogs? A Sub-Continental Assessment of the Chytrid Fungus (Batrachochytrium dendrobatidis). PLoS One. 2013;8 e56236 10.1371/journal.pone.0056236 23426141 PMC3572032

[29] Goldberg TL, Readel AM, Lee MH. Chytrid fungus in frogs from an equatorial African montane forest in western Uganda. J Wildl Dis. 2007;43: 521–524. 17699093 10.7589/0090-3558-43.3.521

[30] Greenbaum E, Kusamba C, Aristote MM, Reed KD. Amphibian chytrid fungus infections in Hyperolius (Anura: Hyperoliidae) from Eastern Democratic Republic of Congo. Herpetol Rev. 2008;39: 70–73.

[31] Greenbaum E, Meece J, Reed KD, Kusamba C. Extensive occurrance of the amphibian chytrid fungus in the Albertine Rift, a Central African amphibian hotspot. Herpetol J. 2015;25: 91–100.

[32] Greenbaum E, Meece J, Reed KD. Amphibian chytrid Infections in non-forested habitats of Katanga, Democratic Republic of the Congo. 2014;45: 610–614.

[33] Soto-Azat C, Clarke BT, Poynton JC, Cunningham A. Widespread historical presence of Batrachochytrium dendrobatidis in African pipid frogs. Divers Distrib. 2010;16: 126–131. 10.1111/j.1472-4642.2009.00618.x

[34] Rohr JR, Raffel TR, Romansic JM, McCallum H, Hudson PJ. Evaluating the links between climate, disease spread, and amphibian declines. Proc Natl Acad Sci U S A. 2008;105: 17436–17441. 10.1073/pnas.0806368105 18987318 PMC2582253

[35] Seimon T, Seimon A, Daszak P, Halloy S. P, Schloegel L, Aguilar C, et al Upward range extension of Andean anurans and chytridiomycosis to extreme elevations in response to tropical deglaciation. Glob Chang Biol. 2007;13: 288–299. 10.1111/j.1365-2486.2006.01278.x

[36] Raxworthy CJ, Pearson RG, Rabibisoa N, Rakotondrazafy AM, Ramanamanjato JB, Raselimanana AP, et al Extinction vulnerability of tropical montane endemism from warming and upslope displacement: A preliminary appraisal for the highest massif in Madagascar. Glob Chang Biol. 2008;14: 1703–1720. 10.1111/j.1365-2486.2008.01596.x

[37] Pounds JA, Fogden MPL, Campbell JH. Biological response to climate change on a tropical mountain. Nature. 1999;398: 611–615.

[38] Catenazzi A, von May R, Vredenburg VT. High prevalence of infection in tadpoles increases vulnerability to fungal pathogen in high-Andean amphibians. Biol Conserv. 2013;159: 413–421. 10.1016/j.biocon.2012.11.023

[39] McMenamin SK, Hadly E a, Wright CK. Climatic change and wetland desiccation cause amphibian decline in Yellowstone National Park. Proc Natl Acad Sci U S A. 2008;105: 16988–16993. 10.1073/pnas.0809090105 18955700 PMC2579365

[40] Seimon A, Picton-Phillipps GP. Regional climatology of the Albertine Rift. Long-term changes in Africa’s Rift Valley. In: Plumptre A, editor. New York: Nova Science Publishers; 2012.

[41] Seimon A, Plumptre A. The Albertine Rift., Conservation and climate disruption: Landscape science and practice in a changing climate. In: Cross M, Hilty J, Chester C, editor. Island Press; 2012.

[42] Seimon A, Ingram C, Watson J. Climatology of the East African Great Lakes Region and potential impacts of climate change on its biodiversity and ecosystem services In: Gordon I, editor. Conservation Strategy in the Great Lakes Region. Chicago; 2013 pp. 103–134.

[43] James TY, Toledo LF, Rödder D, da Silva Leite D, Belasen AM, Betancourt-Román CM, et al Disentangling host, pathogen, and environmental determinants of a recently emerged wildlife disease: lessons from the first 15 years of amphibian chytridiomycosis research. Ecol Evol. 2015;5: 4079–4097. 10.1002/ece3.1672 26445660 PMC4588650

[44] Pessier AP, Mendelson J. R. III. A manual for control of infectious diseases in amphibian survival assurance colonies and reintroduction programs. In: Pessier AP and M JR I, editor. 2009.

[45] Brem F., Mendelson J.R. III, and Lips KR. Field-sampling protocol for Batrachochytrium dendrobatidis from living amphibians, using alcohol preserved awabs. Version 1. Arlington, Virginia, USA: Conservation International; 2007 Available: Version 1.0 http://www.amphibians.org

[46] Greenbaum EJ, Sinsch U, Lehr E, Valdez F, Chifundera Z. Phylogeography of the reed frog Hyperolius castaneus (Anura: Hyperoliidae) from the Albertine Rift of central Africa: Implications for taxonomy, biogeography and conservation. Zootaxa. 2013;3731: 473–494. 10.11646/zootaxa.3731.4.3 25277586

[47] Boyle DG, Boyle DB, Olsen V, Morgan J a T, Hyatt A. D. Rapid quantitative detection of chytridiomycosis (Batrachochytrium dendrobatidis) in amphibian samples using real-time Taqman PCR assay. Dis Aquat Organ. 2004;60: 141–148. 10.3354/dao060141 15460858

[48] Brown L, Cat T, DasGupta A. Interval estimation for a proportion. Stat Sci. 2001;16: 101–133.

[49] Soto-Azat C, Clarke BT, Fisher MC, Walker SF, Cunningham AA. Non-invasive sampling methods for the detection of *Batrachochytrium dendrobatidis* in archived amphibians. Dis Aquat Organ. 2009;84: 163–166. 10.3354/dao02029 19476287

[50] Annis SL, Dastoor FP, Ziel H, Daszak P, Longcore JE. A DNA-based assay identifies *Batrachochytrium dendrobatidis* in amphibians. J Wildl Dis. 2004;40: 420–8. 15465708 10.7589/0090-3558-40.3.420

[51] Phillips SJ, Anderson RP, Schapire RE. Maximum entropy modelling of species geographic distributions. Ecol Modell. 2006;190: 231–259.

[52] Phillips SJ, Dudík M. Modeling of species distributions with Maxent: New extensions and a comprehensive evaluation. Ecography (Cop). 2008;31: 161–175. Available: http://www.cs.princeton.edu/~schapire/maxent/

[53] Elith J, Phillips SJ, Hastie T, Dudık M, Chee YE, Yates CJ. A statistical explanation of MaxEnt for ecologists. Divers Distrib. 2011;17: 43–57.

[54] Phillips SJ, Dudik M, Schapire RE. A maximum entropy approach to species distribution modeling Proceeding of the twenty-first international conference on machine learning. New York: ACM Press, Banff, Canada; 2004 pp. 655–662.

[55] Freeman EA, Moisen G. A comparison of the performance of threshold criteria for binary classification in terms of predicted prevalence and kappa. Ecol Model. 2008;217: 48–58.

[56] Manel S, Williams HC, Ormerod SJ. Evaluating presence–absence models in ecology: the need to account for prevalence. J Appl Ecol. 2001;38: 921–931.

[57] Hijmans RJ, Cameron SE, Parra JL, Jones PG, Jarvis A. Very high resolution interpolated climate surfaces for global land areas. Int J Climatol. 2005;25: 1965–1978.

[58] Stevenson LA, Alford RA, Bell SC, Roznik EA, Berger L, Pike DA. Variation in thermal performance of a widespread pathogen, the amphibian chytrid fungus Batrachochytrium dendrobatidis. PLoS One. 2013;8: 1–14. 10.1371/journal.pone.0073830 PMC3762749 24023908

[59] Muths E, Pilliod DS, Livo LJ. Distribution and environmental limitations of an amphibian pathogen in the Rocky Mountains, USA. Biol Conserv. 2008;141: 1484–1492. 10.1016/j.biocon.2008.03.011

[60] IPCC. Emmissions Scenarios. Nakicenovic N, Swart R, editors. United Kingdom: Cambridge University Press; 2000.

[61] IPCC. Contribution of Working Group I to the Fourth Assessment Report of the Intergovernmental Panel on Climate Change. Solomon S, Qin D, Manning M, Chen Z, Marquis M, Averyt K, et al, editors. Cambridge, United Kingdom and New York, NY, USA.: Cambridge University Press; 2007.

[62] Longo AV., Rodriguez D, da Silva Leite D, Toledo LF, Mendoza Almeralla C, Burrowes P a., et al ITS1 copy number varies among Batrachochytrium dendrobatidis strains: Implications for qPCR estimates of infection intensity from field-collected amphibian skin swabs. PLoS One. 2013;8: 1–10. 10.1371/journal.pone.0059499 PMC3605245 23555682

[63] Goka K, Yokoyama J, Une Y, Kuroki T, Suzuki K, Nakahara M, et al Amphibian chytridiomycosis in Japan: Distribution, haplotypes and possible route of entry into Japan. Mol Ecol. 2009;18: 4757–4774. 10.1111/j.1365-294X.2009.04384.x 19840263

[64] Ron SR. Predicting the distribution of the amphibian pathogen *Batrachochytrium dendrobatidis* in the New World. Biotropica. 2005;37: 209–221.

[65] Hof C, Araújo MB, Jetz W, Rahbek C. Additive threats from pathogens, climate and land-use change for global amphibian diversity. Nature. 2011; 1–6. 10.1038/nature10650 22089134

[66] Bustamante H. M., Livo L. J. and Carey C. Effects of temperature and hydric environment on survival of the Panamanian Golden Frog infected with a pathogenic chytrid fungus. Integr Zool. 2010;5: 143–153. 10.1111/j.1749-4877.2010.00197.x 21392332

[67] Rowley JJL, Alford RA. Hot bodies protect amphibians against chytrid infection in nature. Sci Rep. 2013;3: 1515 10.1038/srep01515 23519020 PMC3604863

[68] Woodhams DC, Alford RA, Marantelli G. Emerging disease of amphibians cured by elevating body temperature. Dis Aquat Organ. 2003;55: 65–67. 10.3354/dao055065 12887256

[69] Berger L, Speare R, Hines HB, Marantelli G, Hyatt AD, McDonald KR, et al Effect of season and temperature on mortality in amphibians due to chytridiomycosis. Aust Vet J. 2004;82: 434–439. 10.1111/j.1751-0813.2004.tb11137.x 15354853

[70] Forrest MJ, Schlaepfer MA. Nothing a hot bath won’t cure: Infection rates of amphibian chytrid fungus correlate negatively with water temperature under natural field settings. PLoS One. 2011;6 e28444 10.1371/journal.pone.0028444 22205950 PMC3244395

[71] Piotrowski JS, Annis SL, Longcore JE. Physiology of *Batrachochytrium dendrobatidis*, a chytrid pathogen of amphibians. Mycologia. 2004;96: 9–15. 10.2307/3761981 21148822

[72] Schloegel LM, Daszak P, Cunningham AA, Speare R, Hill B. Two amphibian diseases, chytridiomycosis and ranaviral disease, are now globally notifiable to the World Organization for Animal Health (OIE): An assessment. Dis Aquat Organ. 2010;92: 101–108. 10.3354/dao02140 21268971

[73] Lane EP, Weldon C, Bingham J. Histological evidence of chytridiomycete fungal infection in a free-ranging amphibian, Afrana fuscigula (Anura: Ranidae), in South Africa : Short communication. Journal of the South African Veterinary Association. 2003 pp. p.20–21. 12836742 10.4102/jsava.v74i1.493

[74] Weldon C, Du Preez LH, Hyatt AD, Muller R, Speare R. Origin of the amphibian chytrid fungus. Emerg Infect Dis. 2004;10: 2100–2105. 10.3201/eid1012.030804 15663845 PMC3323396

[75] Parker JM, Mikaelian I, Hahn N, Diggs HE. Clinical diagnosis and treatment of epidermal chytridiomycosis in African clawed frogs (Xenopus tropicalis). Comp Med. 2002;52: 265–268. 12102573

[76] Daszak P, Strieby A, Cunningham AA, Longcore JE, Brown CC, Porter D. Experimental evidence that the bullfrog (Rana catesbeiana) is a potential carrier of chytridiomycosis, an emerging fungal disease of amphibians. Herpetol J. British Herpetological Society; 2004;14: 201–207.

[77] Gervasi SS, Urbina J, Hua J, Chestnut T, Relyea R.A., Blaustein A.R.. Experimental evidence for American bullfrog (Lithobates catesbeianus) susceptibility to chytrid fungus (Batrachochytrium dendrobatidis). Ecohealth. 2013;10: 166–171. 10.1007/s10393-013-0832-8 23539129

[78] Retallick RWR, Miera V. Strain differences in the amphibian chytrid *Batrachochytrium dendrobatidis* and non-permanent, sub-lethal effects of infection. Dis Aquat Organ. 2007;75: 201–207. 10.3354/dao075201 17629114

[79] Rosenblum EB, James TY, Zamudio KR, Poorten TJ, Ilut D, Rodriguez D, et al Complex history of the amphibian-killing chytrid fungus revealed with genome resequencing data. Proc Natl Acad Sci U S A. 2013;110: 9385–90. 10.1073/pnas.1300130110 23650365 PMC3677446

[80] Rosenblum EB, Fisher MC, James TY, Stajich JE, Longcore JE, Gentry LR, et al A molecular perspective: Biology of the emerging pathogen *Batrachochytrium dendrobatidis* . Dis Aquat Organ. 2010;92: 131–147. 10.3354/dao02179 21268975

[81] Carey C, Bruzgul JE, Livo LJ, Walling ML, Kuehl KA., Dixon BF, et al Experimental exposures of boreal toads (Bufo boreas) to a pathogenic chytrid fungus (Batrachochytrium dendrobatidis). Ecohealth. 2006;3: 5–21.

[82] Rodriguez D, Becker CG, Pupin NC, Haddad CFB, Zamudio KR. Long-term endemism of two highly divergent lineages of the amphibian-killing fungus in the Atlantic forest of Brazil. Mol Ecol. 2014;23: 774–787. 10.1111/mec.12615 24471406

[83] Talley BL, Muletz CR, Vredenburg VT, Fleischer RC, Lips KR. A century of Batrachochytrium dendrobatidis in Illinois amphibians (1888–1989). Biol Conserv. 2015;182: 254–261. 10.1016/j.biocon.2014.12.007

[84] Vredenburg VT, Felt Sa, Morgan EC, McNally SVG, Wilson S, Green SL. Prevalence of Batrachochytrium dendrobatidis in Xenopus collected in Africa (1871–2000) and in California (2001–2010). PLoS One. 2013;8: 6–9. e63791 10.1371/journal.pone.0063791 PMC3655066 23691097

[85] Greenbaum E, Kusamba C. Conservation implications following the rediscovery of four frog species from the Itombwe Natural Reserve, Eastern Democratic Republic of the Congo. Herpetol Rev. 2012;43: 253–259.

[86] Schloegel LM, Toledo LF, Longcore JE, Greenspan SE, Vieira CA, Lee M, et al Novel, panzootic and hybrid genotypes of amphibian chytridiomycosis associated with the bullfrog trade. Mol Ecol. 2012;21: 5162–5177. 10.1111/j.1365-294X.2012.05710.x 22857789

[87] Gilbert M, Bickford D, Clark L, Johnson A, Joyner PH, Ogg Keatts L, et al Amphibian pathogens in southeast asian frog trade. Ecohealth. 2013; 1–13. 10.1007/s10393-013-0817-7 23404036

[88] Herrmann SM, Mohr KI. A continental-scale classification of rainfall seasonality regimes in Africa based on gridded precipitation and land surface temperature products. J Appl Meteor Clim. 2011;50: 2504–2513.

[89] Kriger KM, Hero JM. Large-scale seasonal variation in the prevalence and severity of chytridiomycosis. J Zool. 2007;271: 352–359. 10.1111/j.1469-7998.2006.00220.x

[90] Catenazzi A, Lehr E, Vredenburg VT. Thermal physiology, disease, and amphibian declines on the eastern slopes of the andes. Conserv Biol. 2014;28: 509–517. 10.1111/cobi.12194 24372791

