# Supplementary figures and images for: Assessing the Threat of Amphibian Chytrid Fungus in the Albertine Rift: Past, Present and Future
Source: PLoS One. 2015 Dec 28;10(12):e0145841. doi: 10.1371/journal.pone.0145841 (PMC4692535; doi:10.1371/journal.pone.0145841)

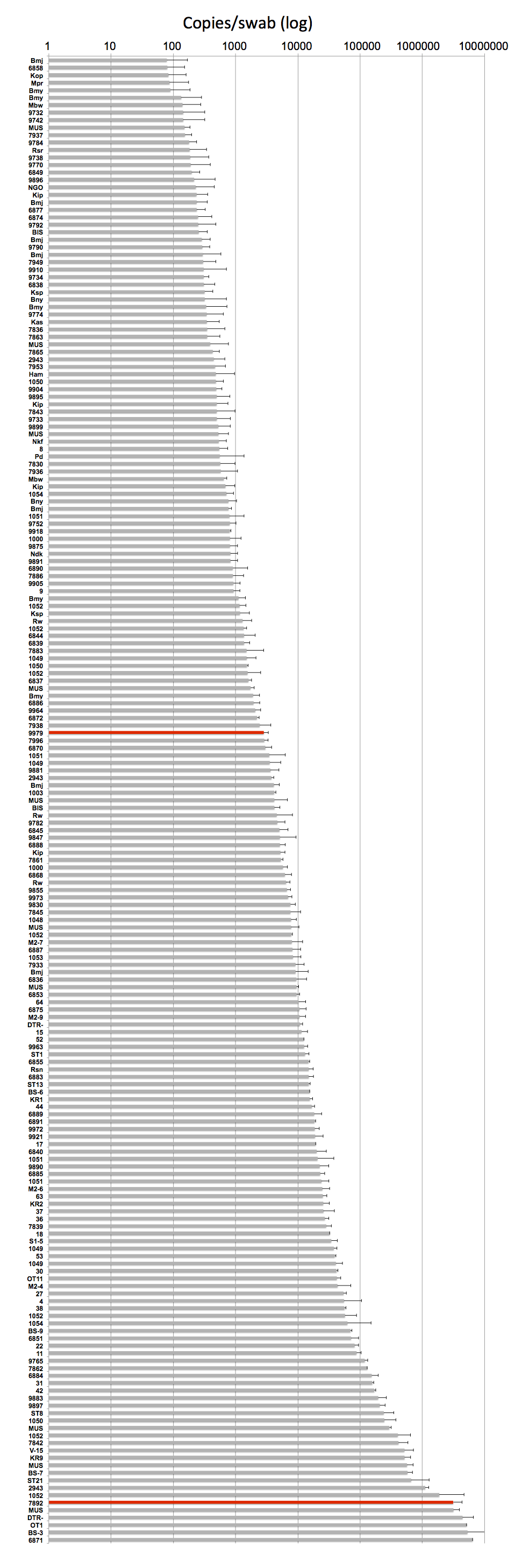

Supplement: S1 Fig — Log-scale graph showing all Bd-positive samples plotted against the calculated number of copies of the ITS1-5.8S region per swab. Error bars indicate the standard deviation (positive only) for each triplicate sample. Red bars indicate which two samples had corresponding skin tissue analyzed and had histological changes consistant with the disease chytridiomycosis. (TIFF) [file pone.0145841.s002.tiff]

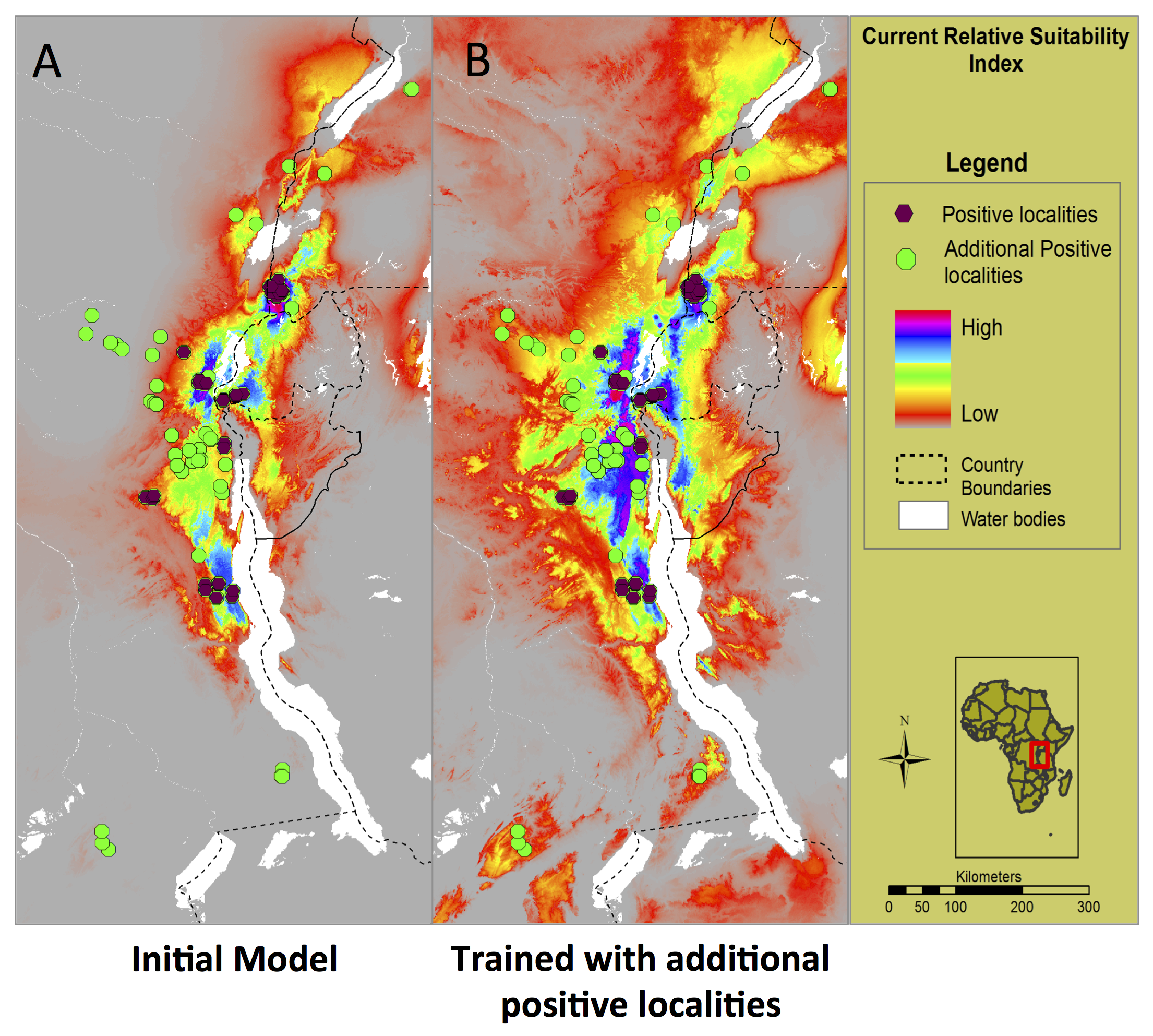

Supplement: S2 Fig — A. (Initial model). Current distribution of areas where amphibians are likely to be at risk for Bd infection using original records (maroon hexagons). All areas predicted as suitable where the new positive localities (green hexagons) appear indicate areas where we had not sampled but the model predicted as a potential suitable habitat. These areas include (Budongo, Kibale, Kamengo, Lake Bunyonyi, North Balala Forest, parts of Itombwe Massif). The model didn’t predict the lowlands of Kahuzi-Biega National Park and Katanga province. B. An updated model showing the current distribution of areas where amphibians are likely to be at risk for Bd infection when using both original (maroon hexagons) and new occurrence (green hexagons) records for training. (TIFF) [file pone.0145841.s003.tiff]
