# Supplementary material for: Assessing the Threat of Amphibian Chytrid Fungus in the Albertine Rift: Past, Present and Future
Source: PLoS One. 2015 Dec 28;10(12):e0145841. doi: 10.1371/journal.pone.0145841 (PMC4692535; doi:10.1371/journal.pone.0145841)
Supplement: S1 Table — Elevations are in meters above sea level. (DOCX) [file pone.0145841.s004.docx]

| **WCS ID** | **RMCA ID** | **Scientific Name (Genus species)** | **Collector** | **Date of Collection** | **Country** | **Location and Elevation** | **Protected area** | **Field Site** | **PCR results** |
| --- | --- | --- | --- | --- | --- | --- | --- | --- | --- |
| AM002 | 106229 | *Hyperolius constellatus** | R. Laurent | 23-30 Sept 1950 | DRC | Ruzizi nr Uvira - 2550m | Itombwe | Haut Luvubu | NEG |
| AM003 | 106228 | *Hyperolius constellatus* | R. Laurent | 23-30 Sept 1950 | DRC | Ruzizi nr Uvira - 2550m | Itombwe | Haut Luvubu | NEG |
| AM004 | 106227 | *Hyperolius constellatus* | R. Laurent | 23-30 Sept 1950 | DRC | Ruzizi nr Uvira - 2550m | Itombwe | Haut Luvubu | NEG |
| AM005 | 106530 | *Hyperolius constellatus* | R. Laurent | 23-30 Sept 1950 | DRC | Ruzizi nr Uvira - 2550m | Itombwe | Haut Luvubu | NEG |
| AM006 | 106529 | *Hyperolius constellatus* | R. Laurent | 23-30 Sept 1950 | DRC | Ruzizi nr Uvira - 2550m | Itombwe | Haut Luvubu | NEG |
| AM007 | 106528 | *Hyperolius constellatus* | R. Laurent | 23-30 Sept 1950 | DRC | Ruzizi nr Uvira - 2550m | Itombwe | Haut Luvubu | NEG |
| AM008 | 106527 | *Hyperolius constellatus* | R. Laurent | 23-30 Sept 1950 | DRC | Ruzizi nr Uvira - 2550m | Itombwe | Haut Luvubu | NEG |
| AM009 | 107080 | *Hyperolius constellatus* | R. Laurent | 23-30 Sept 1950 | DRC | Ruzizi nr Uvira - 2550m | Itombwe | Haut Luvubu | NEG |
| AM010 | 107079 | *Hyperolius constellatus* | R. Laurent | 23-30 Sept 1950 | DRC | Ruzizi nr Uvira - 2550m | Itombwe | Haut Luvubu | NEG |
| AM011 | 107078 | *Hyperolius constellatus* | R. Laurent | 23-30 Sept 1950 | DRC | Ruzizi nr Uvira - 2550m | Itombwe | Haut Luvubu | NEG |
| AM012 | 107077 | *Hyperolius constellatus* | R. Laurent | 23-30 Sept 1950 | DRC | Ruzizi nr Uvira - 2550m | Itombwe | Haut Luvubu | NEG |
| AM013 | 107530 | *Hyperolius constellatus* | R. Laurent | 23-30 Sept 1950 | DRC | Ruzizi nr Uvira - 2550m | Itombwe | Haut Luvubu | INDETERMINATE |
| AM014 | 107527 | *Hyperolius constellatus* | R. Laurent | 23-30 Sept 1950 | DRC | Ruzizi nr Uvira - 2550m | Itombwe | Haut Luvubu | NEG |
| AM015 | 107529 | *Hyperolius constellatus* | R. Laurent | 23-30 Sept 1950 | DRC | Ruzizi nr Uvira - 2550m | Itombwe | Haut Luvubu | NEG |
| AM016 | 107528 | *Hyperolius constellatus* | R. Laurent | 23-30 Sept 1950 | DRC | Ruzizi nr Uvira - 2550m | Itombwe | Haut Luvubu | NEG |
| AM017 | 105466 | *Hyperolius constellatus* | R. Laurent | 28 Sept 1950 | DRC | Bassin de l'Ulindi - 2650m | Itombwe | Marais de Kilungutwe | NEG |
| AM018 | 105465 | *Hyperolius constellatus* | R. Laurent | 28 Sept 1950 | DRC | Bassin de l'Ulindi - 2650m | Itombwe | Marais de Kilungutwe | NEG |
| AM019 | 105464 | *Hyperolius constellatus* | R. Laurent | 28 Sept 1950 | DRC | Bassin de l'Ulindi - 2650m | Itombwe | Marais de Kilungutwe | INDETERMINATE |
| AM020 | 105463 | *Hyperolius constellatus* | R. Laurent | 28 Sept 1950 | DRC | Bassin de l'Ulindi - 2650m | Itombwe | Marais de Kilungutwe | NEG |
| AM021 | 115260 | *Leptopelis karissimbiensis* | R. Laurent | 18-21 Jan 1951 | Rwanda | Gisenyi territory - 2400m | Bwindi-Virunga | Mwyaniki Lake | NEG |
| AM022 | 115259 | *Leptopelis karissimbiensis* | R. Laurent | 18-21 Jan 1951 | Rwanda | Gisenyi territory - 2400m | Bwindi-Virunga | Mwyaniki Lake | NEG |
| AM023 | 115258 | *Leptopelis karissimbiensis* | R. Laurent | 18-21 Jan 1951 | Rwanda | Gisenyi territory - 2400m | Bwindi-Virunga | Mwyaniki Lake | NEG |
| AM024 | 115257 | *Leptopelis karissimbiensis* | R. Laurent | 18-21 Jan 1951 | Rwanda | Gisenyi territory - 2400m | Bwindi-Virunga | Mwyaniki Lake | NEG |
| AM025 | 113469 | *Leptopelis karissimbiensis* | R. Laurent | 25-26 Jul 1952 | Uganda | Nyakaroro - terr of Muramuya - 2250m | Bwindi | River Mubarazi | NEG |
| AM026 | 113468 | *Leptopelis karissimbiensis* | R. Laurent | 25-26 Jul 1952 | Uganda | Nyakaroro - terr of Muramuya - 2250m | Bwindi | River Mubarazi | NEG |
| AM027 | 113467 | *Leptopelis karissimbiensis* | R. Laurent | 25-26 Jul 1952 | Uganda | Nyakaroro - terr of Muramuya - 2250m | Bwindi | River Mubarazi | NEG |
| AM028 | 113466 | *Leptopelis karissimbiensis* | R. Laurent | 25-26 Jul 1952 | Uganda | Nyakaroro - terr of Muramuya - 2250m | Bwindi | River Mubarazi | NEG |
| AM029 | 113465 | *Leptopelis karissimbiensis* | R. Laurent | 25-26 Jul 1952 | Uganda | Nyakaroro - terr of Muramuya - 2250m | Bwindi | River Mubarazi | NEG |
| AM030 | 113464 | *Leptopelis karissimbiensis* | R. Laurent | 25-26 Jul 1952 | Uganda | Nyakaroro - terr of Muramuya - 2250m | Bwindi | River Mubarazi | NEG |
| AM031 | 113338 | *Leptopelis karissimbiensis* | R. Laurent | 28-29 Jan 1951 | Rwanda | Ruhengeri terr - 2100m | Bwindi-Virunga | Rusumu marais | NEG |
| AM032 | 113337 | *Leptopelis karissimbiensis* | R. Laurent | 28-29 Jan 1951 | Rwanda | Ruhengeri terr - 2100m | Bwindi-Virunga | Rusumu marais | NEG |
| AM033 | 113336 | *Leptopelis karissimbiensis* | R. Laurent | 28-29 Jan 1951 | Rwanda | Ruhengeri terr - 2100m | Bwindi-Virunga | Rusumu marais | NEG |
| AM034 | 113335 | *Leptopelis karissimbiensis* | R. Laurent | 28-29 Jan 1951 | Rwanda | Ruhengeri terr - 2100m | Bwindi-Virunga | Rusumu marais | NEG |
| AM035 | 113334 | *Leptopelis karissimbiensis* | R. Laurent | 28-29 Jan 1951 | Rwanda | Ruhengeri terr - 2100m | Bwindi-Virunga | Rusumu marais | NEG |
| AM036 | 113333 | *Leptopelis karissimbiensis* | R. Laurent | 28-29 Jan 1951 | Rwanda | Ruhengeri terr - 2100m | Bwindi-Virunga | Rusumu marais | NEG |
| AM037 | 115278 | *Leptopelis karissimbiensis* | R. Laurent | 27-29 Jan 1951 | Rwanda | Ruhengeri terr - 1870-1900m | Bwindi-Virunga | Lac Bulera | NEG |
| AM038 | 115277 | *Leptopelis karissimbiensis* | R. Laurent | 27-29 Jan 1951 | Rwanda | Ruhengeri terr - 1870-1900m | Bwindi-Virunga | Lac Bulera | NEG |
| AM039 | 115276 | *Leptopelis karissimbiensis* | R. Laurent | 27-29 Jan 1951 | Rwanda | Ruhengeri terr - 1870-1900m | Bwindi-Virunga | Lac Bulera | NEG |
| AM040 | 115275 | *Leptopelis karissimbiensis* | R. Laurent | 27-29 Jan 1951 | Rwanda | Ruhengeri terr - 1870-1900m | Bwindi-Virunga | Lac Bulera | NEG |
| AM041 | 114089 | *Leptopelis kivuensis* | R. Laurent | 28 May 1949 | DRC | Kalehe Terr - 2200m | Kahuzi Biega | River Tshinia | INDETERMINATE |
| AM042 | 114088 | *Leptopelis kivuensis* | R. Laurent | 28 May 1949 | DRC | Kalehe Terr - 2200m | Kahuzi Biega | River Tshinia | NEG |
| AM043 | 114087 | *Leptopelis kivuensis* | R. Laurent | 28 May 1949 | DRC | Kalehe Terr - 2200m | Kahuzi Biega | River Tshinia | INDETERMINATE |
| AM044 | 114086 | *Leptopelis kivuensis* | R. Laurent | 28 May 1949 | DRC | Kalehe Terr - 2200m | Kahuzi Biega | River Tshinia | NEG |
| AM045 | 114085 | *Leptopelis kivuensis* | R. Laurent | 28 May 1949 | DRC | Kalehe Terr - 2200m | Kahuzi Biega | River Tshinia | NEG |
| AM046 | 114084 | *Leptopelis kivuensis* | R. Laurent | 28 May 1949 | DRC | Kalehe Terr - 2200m | Kahuzi Biega | River Tshinia | NEG |
| AM047 | 114083 | *Leptopelis kivuensis* | R. Laurent | 28 May 1949 | DRC | Kalehe Terr - 2200m | Kahuzi Biega | River Tshinia | NEG |
| AM048 | 114082 | *Leptopelis kivuensis* | R. Laurent | 28 May 1949 | DRC | Kalehe Terr - 2200m | Kahuzi Biega | River Tshinia | NEG |
| AM049 | 114081 | *Leptopelis kivuensis* | R. Laurent | 28 May 1949 | DRC | Kalehe Terr - 2200m | Kahuzi Biega | River Tshinia | NEG |
| AM050 | 114080 | *Leptopelis kivuensis* | R. Laurent | 28 May 1949 | DRC | Kalehe Terr - 2200m | Kahuzi Biega | River Tshinia | NEG |
| AM051 | 114079 | *Leptopelis kivuensis* | R. Laurent | 28 May 1949 | DRC | Kalehe Terr - 2200m | Kahuzi Biega | River Tshinia | INDETERMINATE |
| AM052 | 114078 | *Leptopelis kivuensis* | R. Laurent | 28 May 1949 | DRC | Kalehe Terr - 2200m | Kahuzi Biega | River Tshinia | NEG |
| AM053 | 114077 | *Leptopelis kivuensis* | R. Laurent | 28 May 1949 | DRC | Kalehe Terr - 2200m | Kahuzi Biega | River Tshinia | INDETERMINATE |
| AM054 | 114076 | *Leptopelis kivuensis* | R. Laurent | 28 May 1949 | DRC | Kalehe Terr - 2200m | Kahuzi Biega | River Tshinia | NEG |
| AM055 | 114075 | *Leptopelis kivuensis* | R. Laurent | 28 May 1949 | DRC | Kalehe Terr - 2200m | Kahuzi Biega | River Tshinia | INDETERMINATE |
| AM056 | 114074 | *Leptopelis kivuensis* | R. Laurent | 28 May 1949 | DRC | Kalehe Terr - 2200m | Kahuzi Biega | River Tshinia | INDETERMINATE |
| AM057 | 114073 | *Leptopelis kivuensis* | R. Laurent | 28 May 1949 | DRC | Kalehe Terr - 2200m | Kahuzi Biega | River Tshinia | NEG |
| AM058 | 114072 | *Leptopelis kivuensis* | R. Laurent | 28 May 1949 | DRC | Kalehe Terr - 2200m | Kahuzi Biega | River Tshinia | NEG |
| AM059 | 114071 | *Leptopelis kivuensis* | R. Laurent | 28 May 1949 | DRC | Kalehe Terr - 2200m | Kahuzi Biega | River Tshinia | NEG |
| AM060 | 114070 | *Leptopelis kivuensis* | R. Laurent | 28 May 1949 | DRC | Kalehe Terr - 2200m | Kahuzi Biega | River Tshinia | NEG |
| AM061 | 114060 | *Leptopelis kivuensis* | R. Laurent | 18-19 July 1949 | DRC | Mwenga Terr - 2000m | Itombwe | Miki | NEG |
| AM062 | 114059 | *Leptopelis kivuensis* | R. Laurent | 18-19 July 1949 | DRC | Mwenga Terr - 2000m | Itombwe | Miki | NEG |
| AM063 | 114058 | *Leptopelis kivuensis* | R. Laurent | 18-19 July 1949 | DRC | Mwenga Terr - 2000m | Itombwe | Miki | INDETERMINATE |
| AM064 | 114057 | *Leptopelis kivuensis* | R. Laurent | 18-19 July 1949 | DRC | Mwenga Terr - 2000m | Itombwe | Miki | NEG |
| AM065 | 114056 | *Leptopelis kivuensis* | R. Laurent | 18-19 July 1949 | DRC | Mwenga Terr - 2000m | Itombwe | Miki | NEG |
| AM066 | 114055 | *Leptopelis kivuensis* | R. Laurent | 18-19 July 1949 | DRC | Mwenga Terr - 2000m | Itombwe | Miki | NEG |
| AM067 | 114054 | *Leptopelis kivuensis* | R. Laurent | 18-19 July 1949 | DRC | Mwenga Terr - 2000m | Itombwe | Miki | NEG |
| AM068 | 114151 | *Leptopelis kivuensis* | R. Laurent | 12-16 July 1949 | DRC | Mwenga Terr - 2200-2300m | Itombwe | Kabilombo | INDETERMINATE |
| AM069 | 114150 | *Leptopelis kivuensis* | R. Laurent | 12-16 July 1949 | DRC | Mwenga Terr - 2200-2300m | Itombwe | Kabilombo | INDETERMINATE |
| AM070 | 114149 | *Leptopelis kivuensis* | R. Laurent | 12-16 July 1949 | DRC | Mwenga Terr - 2200-2300m | Itombwe | Kabilombo | NEG |
| AM071 | 114148 | *Leptopelis kivuensis* | R. Laurent | 12-16 July 1949 | DRC | Mwenga Terr - 2200-2300m | Itombwe | Kabilombo | NEG |
| AM072 | 114147 | *Leptopelis kivuensis* | R. Laurent | 12-16 July 1949 | DRC | Mwenga Terr - 2200-2300m | Itombwe | Kabilombo | NEG |
| AM073 | 114146 | *Leptopelis kivuensis* | R. Laurent | 12-16 July 1949 | DRC | Mwenga Terr - 2200-2300m | Itombwe | Kabilombo | NEG |
| AM074 | 114145 | *Leptopelis kivuensis* | R. Laurent | 12-16 July 1949 | DRC | Mwenga Terr - 2200-2300m | Itombwe | Kabilombo | NEG |
| AM075 | 114229 | *Leptopelis kivuensis* | R. Laurent | 20-22 July 1949 | DRC | Mwenga Terr - 1850-1950m | Itombwe | Kiandjo | INDETERMINATE |
| AM076 | 114228 | *Leptopelis kivuensis* | R. Laurent | 20-22 July 1949 | DRC | Mwenga Terr - 1850-1950m | Itombwe | Kiandjo | NEG |
| AM077 | 114227 | *Leptopelis kivuensis* | R. Laurent | 20-22 July 1949 | DRC | Mwenga Terr - 1850-1950m | Itombwe | Kiandjo | NEG |
| AM078 | 114226 | *Leptopelis kivuensis* | R. Laurent | 20-22 July 1949 | DRC | Mwenga Terr - 1850-1950m | Itombwe | Kiandjo | NEG |
| AM079 | 114225 | *Leptopelis kivuensis* | R. Laurent | 20-22 July 1949 | DRC | Mwenga Terr - 1850-1950m | Itombwe | Kiandjo | NEG |
| AM080 | 114224 | *Leptopelis kivuensis* | R. Laurent | 20-22 July 1949 | DRC | Mwenga Terr - 1850-1950m | Itombwe | Kiandjo | NEG |
| AM081 | 114223 | *Leptopelis kivuensis* | R. Laurent | 20-22 July 1949 | DRC | Mwenga Terr - 1850-1950m | Itombwe | Kiandjo | NEG |
| AM082 | 114550 | *Leptopelis kivuensis* | R. Laurent | 20-27 June 1951 | DRC | Mwenga Terr - 2300m | Itombwe | Bubutubu | NEG |
| AM083 | 114549 | *Leptopelis kivuensis* | R. Laurent | 20-27 June 1951 | DRC | Mwenga Terr - 2300m | Itombwe | Bubutubu | NEG |
| AM084 | 114548 | *Leptopelis kivuensis* | R. Laurent | 20-27 June 1951 | DRC | Mwenga Terr - 2300m | Itombwe | Bubutubu | INDETERMINATE |
| AM085 | 114547 | *Leptopelis kivuensis* | R. Laurent | 20-27 June 1951 | DRC | Mwenga Terr - 2300m | Itombwe | Bubutubu | NEG |
| AM086 | 114546 | *Leptopelis kivuensis* | R. Laurent | 20-27 June 1951 | DRC | Mwenga Terr - 2300m | Itombwe | Bubutubu | NEG |
| AM087 | 114545 | *Leptopelis kivuensis* | R. Laurent | 20-27 June 1951 | DRC | Mwenga Terr - 2300m | Itombwe | Bubutubu | NEG |
| AM088 | 114544 | *Leptopelis kivuensis* | R. Laurent | 20-27 June 1951 | DRC | Mwenga Terr - 2300m | Itombwe | Bubutubu | NEG |
| AM089 | 114543 | *Leptopelis kivuensis* | R. Laurent | 20-27 June 1951 | DRC | Mwenga Terr - 2300m | Itombwe | Bubutubu | NEG |
| AM090 | 114542 | *Leptopelis kivuensis* | R. Laurent | 20-27 June 1951 | DRC | Mwenga Terr - 2300m | Itombwe | Bubutubu | NEG |
| AM091 | 114541 | *Leptopelis kivuensis* | R. Laurent | 20-27 June 1951 | DRC | Mwenga Terr - 2300m | Itombwe | Bubutubu | NEG |
| AM092 | 114811 | *Leptopelis kivuensis* | R. Laurent | 28-29 Aug 1950 | DRC | Uvira Terr - 2100-2200m | Itombwe | Lubuka | NEG |
| AM093 | 114810 | *Leptopelis kivuensis* | R. Laurent | 28-29 Aug 1950 | DRC | Uvira Terr - 2100-2200m | Itombwe | Lubuka | NEG |
| AM094 | 114809 | *Leptopelis kivuensis* | R. Laurent | 28-29 Aug 1950 | DRC | Uvira Terr - 2100-2200m | Itombwe | Lubuka | INDETERMINATE |
| AM095 | 114808 | *Leptopelis kivuensis* | R. Laurent | 28-29 Aug 1950 | DRC | Uvira Terr - 2100-2200m | Itombwe | Lubuka | NEG |
| AM096 | 114807 | *Leptopelis kivuensis* | R. Laurent | 28-29 Aug 1950 | DRC | Uvira Terr - 2100-2200m | Itombwe | Lubuka | NEG |
| AM097 | 113501 | *Leptopelis kivuensis* | R. Laurent | 25-27 Aug 1950 | DRC | Mwenga Terr. - 1650m | Itombwe | Mwana | NEG |
| AM098 | 113500 | *Leptopelis kivuensis* | R. Laurent | 25-27 Aug 1950 | DRC | Mwenga Terr. - 1650m | Itombwe | Mwana | NEG |
| AM099 | 113499 | *Leptopelis kivuensis* | R. Laurent | 25-27 Aug 1950 | DRC | Mwenga Terr. - 1650m | Itombwe | Mwana | NEG |
| AM100 | 113498 | *Leptopelis kivuensis* | R. Laurent | 25-27 Aug 1950 | DRC | Mwenga Terr. - 1650m | Itombwe | Mwana | NEG |
| AM101 | 113497 | *Leptopelis kivuensis* | R. Laurent | 25-27 Aug 1950 | DRC | Mwenga Terr. - 1650m | Itombwe | Mwana | NEG |
| AM102 | 113724 | *Leptopelis kivuensis* | R. Laurent | 11-17 Aug 1949 | Burundi | Ngozi Terr. - 2200m | Nyungwe-Kibira | Muhokole River | NEG |
| AM103 | 113723 | *Leptopelis kivuensis* | R. Laurent | 11-17 Aug 1949 | Burundi | Ngozi Terr. - 2200m | Nyungwe-Kibira | Muhokole River | NEG |
| AM104 | 113722 | *Leptopelis kivuensis* | R. Laurent | 11-17 Aug 1949 | Burundi | Ngozi Terr. - 2200m | Nyungwe-Kibira | Muhokole River | NEG |
| AM105 | 113721 | *Leptopelis kivuensis* | R. Laurent | 11-17 Aug 1949 | Burundi | Ngozi Terr. - 2200m | Nyungwe-Kibira | Muhokole River | NEG |
| AM106 | 113720 | *Leptopelis kivuensis* | R. Laurent | 11-17 Aug 1949 | Burundi | Ngozi Terr. - 2200m | Nyungwe-Kibira | Muhokole River | NEG |
| AM107 | 113719 | *Leptopelis kivuensis* | R. Laurent | 11-17 Aug 1949 | Burundi | Ngozi Terr. - 2200m | Nyungwe-Kibira | Muhokole River | INDETERMINATE |
| AM108 | 113718 | *Leptopelis kivuensis* | R. Laurent | 11-17 Aug 1949 | Burundi | Ngozi Terr. - 2200m | Nyungwe-Kibira | Muhokole River | NEG |
| AM109 | 113717 | *Leptopelis kivuensis* | R. Laurent | 11-17 Aug 1949 | Burundi | Ngozi Terr. - 2200m | Nyungwe-Kibira | Muhokole River | INDETERMINATE |
| AM110 | 113716 | *Leptopelis kivuensis* | R. Laurent | 11-17 Aug 1949 | Burundi | Ngozi Terr. - 2200m | Nyungwe-Kibira | Muhokole River | INDETERMINATE |
| AM111 | 113715 | *Leptopelis kivuensis* | R. Laurent | 11-17 Aug 1949 | Burundi | Ngozi Terr. - 2200m | Nyungwe-Kibira | Muhokole River | NEG |
| AM112 | 113714 | *Leptopelis kivuensis* | R. Laurent | 11-17 Aug 1949 | Burundi | Ngozi Terr. - 2200m | Nyungwe-Kibira | Muhokole River | NEG |
| AM113 | 113713 | *Leptopelis kivuensis* | R. Laurent | 11-17 Aug 1949 | Burundi | Ngozi Terr. - 2200m | Nyungwe-Kibira | Muhokole River | INDETERMINATE |
| AM114 | 113712 | *Leptopelis kivuensis* | R. Laurent | 11-17 Aug 1949 | Burundi | Ngozi Terr. - 2200m | Nyungwe-Kibira | Muhokole River | NEG |
| AM115 | 113711 | *Leptopelis kivuensis* | R. Laurent | 11-17 Aug 1949 | Burundi | Ngozi Terr. - 2200m | Nyungwe-Kibira | Muhokole River | NEG |
| AM116 | 113710 | *Leptopelis kivuensis* | R. Laurent | 11-17 Aug 1949 | Burundi | Ngozi Terr. - 2200m | Nyungwe-Kibira | Muhokole River | INDETERMINATE |
| AM117 | 113709 | *Leptopelis kivuensis* | R. Laurent | 11-17 Aug 1949 | Burundi | Ngozi Terr. - 2200m | Nyungwe-Kibira | Muhokole River | INDETERMINATE |
| AM118 | 113708 | *Leptopelis kivuensis* | R. Laurent | 11-17 Aug 1949 | Burundi | Ngozi Terr. - 2200m | Nyungwe-Kibira | Muhokole River | NEG |
| AM119 | 113707 | *Leptopelis kivuensis* | R. Laurent | 11-17 Aug 1949 | Burundi | Ngozi Terr. - 2200m | Nyungwe-Kibira | Muhokole River | NEG |
| AM120 | 113706 | *Leptopelis kivuensis* | R. Laurent | 11-17 Aug 1949 | Burundi | Ngozi Terr. - 2200m | Nyungwe-Kibira | Muhokole River | NEG |
| AM121 | 113705 | *Leptopelis kivuensis* | R. Laurent | 11-17 Aug 1949 | Burundi | Ngozi Terr. - 2200m | Nyungwe-Kibira | Muhokole River | INDETERMINATE |
| AM122 | 108886 | *Phrynobatrachus asper* | R. Laurent | 2-8 Aug 1950 | DRC | Mwenga Terr. - 2450-2500m | Itombwe | River Makenda | NEG |
| AM123 | 108885 | *Phrynobatrachus asper* | R. Laurent | 2-8 Aug 1950 | DRC | Mwenga Terr. - 2450-2500m | Itombwe | River Makenda | NEG |
| AM124 | 108884 | *Phrynobatrachus asper* | R. Laurent | 2-8 Aug 1950 | DRC | Mwenga Terr. - 2450-2500m | Itombwe | River Makenda | NEG |
| AM125 | 108883 | *Phrynobatrachus asper* | R. Laurent | 2-8 Aug 1950 | DRC | Mwenga Terr. - 2450-2500m | Itombwe | River Makenda | NEG |
| AM126 | 108882 | *Phrynobatrachus asper* | R. Laurent | 2-8 Aug 1950 | DRC | Mwenga Terr. - 2450-2500m | Itombwe | River Makenda | NEG |
| AM127 | 108881 | *Phrynobatrachus asper* | R. Laurent | 2-8 Aug 1950 | DRC | Mwenga Terr. - 2450-2500m | Itombwe | River Makenda | NEG |
| AM128 | 108880 | *Phrynobatrachus asper* | R. Laurent | 2-8 Aug 1950 | DRC | Mwenga Terr. - 2450-2500m | Itombwe | River Makenda | NEG |
| AM129 | 108879 | *Phrynobatrachus asper* | R. Laurent | 2-8 Aug 1950 | DRC | Mwenga Terr. - 2450-2500m | Itombwe | River Makenda | NEG |
| AM130 | 108878 | *Phrynobatrachus asper* | R. Laurent | 2-8 Aug 1950 | DRC | Mwenga Terr. - 2450-2500m | Itombwe | River Makenda | **POS** |
| AM131 | 108877 | *Phrynobatrachus asper* | R. Laurent | 2-8 Aug 1950 | DRC | Mwenga Terr. - 2450-2500m | Itombwe | River Makenda | NEG |
| AM132 | 108902 | *Phrynobatrachus asper* | R. Laurent | 28 Sep 1950 | DRC | Uvira Terr - 2650m | Itombwe | Kilungutwe marais | NEG |
| AM133 | 108901 | *Phrynobatrachus asper* | R. Laurent | 28 Sep 1950 | DRC | Uvira Terr - 2650m | Itombwe | Kilungutwe marais | NEG |
| AM134 | 108900 | *Phrynobatrachus asper* | R. Laurent | 28 Sep 1950 | DRC | Uvira Terr - 2650m | Itombwe | Kilungutwe marais | NEG |
| AM135 | 108899 | *Phrynobatrachus asper* | R. Laurent | 28 Sep 1950 | DRC | Uvira Terr - 2650m | Itombwe | Kilungutwe marais | NEG |
| AM136 | 108898 | *Phrynobatrachus asper* | R. Laurent | 28 Sep 1950 | DRC | Uvira Terr - 2650m | Itombwe | Kilungutwe marais | NEG |
| AM137 | 108922 | *Phrynobatrachus asper* | R. Laurent | 4-8 Sep 1950 | DRC | Uvira Terr. - 2800m | Itombwe | Haute Sanghe | NEG |
| AM138 | 108921 | *Phrynobatrachus asper* | R. Laurent | 4-8 Sep 1950 | DRC | Uvira Terr. - 2800m | Itombwe | Haute Sanghe | INDETERMINATE |
| AM139 | 108920 | *Phrynobatrachus asper* | R. Laurent | 4-8 Sep 1950 | DRC | Uvira Terr. - 2800m | Itombwe | Haute Sanghe | INDETERMINATE |
| AM140 | 108919 | *Phrynobatrachus asper* | R. Laurent | 4-8 Sep 1950 | DRC | Uvira Terr. - 2800m | Itombwe | Haute Sanghe | INDETERMINATE |
| AM141 | 108918 | *Phrynobatrachus asper* | R. Laurent | 4-8 Sep 1950 | DRC | Uvira Terr. - 2800m | Itombwe | Haute Sanghe | NEG |
| AM142 | 1798 | *Phrynobatrachus versicolor* | Schouteden | Jun-Jul 1925 | DRC | No alt given | Virunga | Lulenga | NEG |
| AM143 | 1797 | *Phrynobatrachus versicolor* | Schouteden | Jun-Jul 1925 | DRC | No alt given | Virunga | Lulenga | NEG |
| AM144 | 1796 | *Phrynobatrachus versicolor* | Schouteden | Jun-Jul 1925 | DRC | No alt given | Virunga | Lulenga | NEG |
| AM145 | 1795 | *Phrynobatrachus versicolor* | Schouteden | Jun-Jul 1925 | DRC | No alt given | Virunga | Lulenga | NEG |
| AM146 | 1794 | *Phrynobatrachus versicolor* | Schouteden | Jun-Jul 1925 | DRC | No alt given | Virunga | Lulenga | NEG |
| AM147 | 1793 | *Phrynobatrachus versicolor* | Schouteden | Jun-Jul 1925 | DRC | No alt given | Virunga | Lulenga | NEG |
| AM148 | 1792 | *Phrynobatrachus versicolor* | Schouteden | Jun-Jul 1925 | DRC | No alt given | Virunga | Lulenga | NEG |
| AM149 | 1791 | *Phrynobatrachus versicolor* | Schouteden | Jun-Jul 1925 | DRC | No alt given | Virunga | Lulenga | NEG |
| AM150 | 1790 | *Phrynobatrachus versicolor* | Schouteden | Jun-Jul 1925 | DRC | No alt given | Virunga | Lulenga | NEG |
| AM151 | 1789 | *Phrynobatrachus versicolor* | Schouteden | Jun-Jul 1925 | DRC | No alt given | Virunga | Lulenga | NEG |
| AM152 | 1788 | *Phrynobatrachus versicolor* | Schouteden | Jun-Jul 1925 | DRC | No alt given | Virunga | Lulenga | NEG |
| AM153 | 1787 | *Phrynobatrachus versicolor* | Schouteden | Jun-Jul 1925 | DRC | No alt given | Virunga | Lulenga | NEG |
| AM154 | 1786 | *Phrynobatrachus versicolor* | Schouteden | Jun-Jul 1925 | DRC | No alt given | Virunga | Lulenga | NEG |
| AM155 | 1785 | *Phrynobatrachus versicolor* | Schouteden | Jun-Jul 1925 | DRC | No alt given | Virunga | Lulenga | NEG |
| AM156 | 1784 | *Phrynobatrachus versicolor* | Schouteden | Jun-Jul 1925 | DRC | No alt given | Virunga | Lulenga | NEG |
| AM157 | 1783 | *Phrynobatrachus versicolor* | Schouteden | Jun-Jul 1925 | DRC | No alt given | Virunga | Lulenga | NEG |
| AM158 | 1782 | *Phrynobatrachus versicolor* | Schouteden | Jun-Jul 1925 | DRC | No alt given | Virunga | Lulenga | NEG |
| AM159 | 1781 | *Phrynobatrachus versicolor* | Schouteden | Jun-Jul 1925 | DRC | No alt given | Virunga | Lulenga | NEG |
| AM160 | 1780 | *Phrynobatrachus versicolor* | Schouteden | Jun-Jul 1925 | DRC | No alt given | Virunga | Lulenga | NEG |
| AM161 | 1779 | *Phrynobatrachus versicolor* | Schouteden | Jun-Jul 1925 | DRC | No alt given | Virunga | Lulenga | NEG |
| AM162 | 49,173 | *Leptopelis karissimbiensis* | G. De Witte | May 1934 | DRC | Nyabikumba - 2226m | Virunga | Marais de Kibere | NEG |
| AM163 | 49,172 | *Leptopelis karissimbiensis* | G. De Witte | May 1934 | DRC | Nyabikumba - 2226m | Virunga | Marais de Kibere | NEG |
| AM164 | 49,171 | *Leptopelis karissimbiensis* | G. De Witte | May 1934 | DRC | Nyabikumba - 2226m | Virunga | Marais de Kibere | NEG |
| AM165 | 49,170 | *Leptopelis karissimbiensis* | G. De Witte | May 1934 | DRC | Nyabikumba - 2226m | Virunga | Marais de Kibere | NEG |
| AM166 | 49,169 | *Leptopelis karissimbiensis* | G. De Witte | May 1934 | DRC | Nyabikumba - 2226m | Virunga | Marais de Kibere | NEG |
| AM167 | 49,168 | *Leptopelis karissimbiensis* | G. De Witte | May 1934 | DRC | Nyabikumba - 2226m | Virunga | Marais de Kibere | NEG |
| AM168 | 49,167 | *Leptopelis karissimbiensis* | G. De Witte | May 1934 | DRC | Nyabikumba - 2226m | Virunga | Marais de Kibere | NEG |
| AM169 | 49,166 | *Leptopelis karissimbiensis* | G. De Witte | May 1934 | DRC | Nyabikumba - 2226m | Virunga | Marais de Kibere | NEG |
| AM170 | 49,165 | *Leptopelis karissimbiensis* | G. De Witte | May 1934 | DRC | Nyabikumba - 2226m | Virunga | Marais de Kibere | NEG |
| AM171 | 49,164 | *Leptopelis karissimbiensis* | G. De Witte | May 1934 | DRC | Nyabikumba - 2226m | Virunga | Marais de Kibere | NEG |
| AM172 | 50,396 | *Leptopelis karissimbiensis* | G. De Witte | Dec 1934 | DRC | Nyiragongo - 2300m | Virunga | Nyamushwe - Biviro | INDETERMINATE |
| AM173 | 50,395 | *Leptopelis karissimbiensis* | G. De Witte | Dec 1934 | DRC | Nyiragongo - 2300m | Virunga | Nyamushwe - Biviro | NEG |
| AM174 | 50,394 | *Leptopelis karissimbiensis* | G. De Witte | Dec 1934 | DRC | Nyiragongo - 2300m | Virunga | Nyamushwe - Biviro | NEG |
| AM175 | 50,393 | *Leptopelis karissimbiensis* | G. De Witte | Dec 1934 | DRC | Nyiragongo - 2300m | Virunga | Nyamushwe - Biviro | NEG |
| AM176 | 50,392 | *Leptopelis karissimbiensis* | G. De Witte | Dec 1934 | DRC | Nyiragongo - 2300m | Virunga | Nyamushwe - Biviro | NEG |
| AM177 | 50,391 | *Leptopelis karissimbiensis* | G. De Witte | Dec 1934 | DRC | Nyiragongo - 2300m | Virunga | Nyamushwe - Biviro | NEG |
| AM178 | 50,390 | *Leptopelis karissimbiensis* | G. De Witte | Dec 1934 | DRC | Nyiragongo - 2300m | Virunga | Nyamushwe - Biviro | NEG |
| AM179 | 50,389 | *Leptopelis karissimbiensis* | G. De Witte | Dec 1934 | DRC | Nyiragongo - 2300m | Virunga | Nyamushwe - Biviro | NEG |
| AM180 | 50,388 | *Leptopelis karissimbiensis* | G. De Witte | Dec 1934 | DRC | Nyiragongo - 2300m | Virunga | Nyamushwe - Biviro | NEG |
| AM181 | 50,387 | *Leptopelis karissimbiensis* | G. De Witte | Dec 1934 | DRC | Nyiragongo - 2300m | Virunga | Nyamushwe - Biviro | NEG |
| AM182 | 42,848 | *Phrynobatrachus versicolor* | G. De Witte | 26 Feb -6 Mar 1934 | DRC | 2000m | Virunga | Lac Magera | NEG |
| AM183 | 42,847 | *Phrynobatrachus versicolor* | G. De Witte | 26 Feb -6 Mar 1934 | DRC | 2000m | Virunga | Lac Magera | NEG |
| AM184 | 42,846 | *Phrynobatrachus versicolor* | G. De Witte | 26 Feb -6 Mar 1934 | DRC | 2000m | Virunga | Lac Magera | NEG |
| AM185 | 42,845 | *Phrynobatrachus versicolor* | G. De Witte | 26 Feb -6 Mar 1934 | DRC | 2000m | Virunga | Lac Magera | NEG |
| AM186 | 42,844 | *Phrynobatrachus versicolor* | G. De Witte | 26 Feb -6 Mar 1934 | DRC | 2000m | Virunga | Lac Magera | NEG |
| AM187 | 42,843 | *Phrynobatrachus versicolor* | G. De Witte | 26 Feb -6 Mar 1934 | DRC | 2000m | Virunga | Lac Magera | NEG |
| AM188 | 42,842 | *Phrynobatrachus versicolor* | G. De Witte | 26 Feb -6 Mar 1934 | DRC | 2000m | Virunga | Lac Magera | NEG |
| AM189 | 42,841 | *Phrynobatrachus versicolor* | G. De Witte | 26 Feb -6 Mar 1934 | DRC | 2000m | Virunga | Lac Magera | NEG |
| AM190 | 42,840 | *Phrynobatrachus versicolor* | G. De Witte | 26 Feb -6 Mar 1934 | DRC | 2000m | Virunga | Lac Magera | NEG |
| AM191 | 42,839 | *Phrynobatrachus versicolor* | G. De Witte | 26 Feb -6 Mar 1934 | DRC | 2000m | Virunga | Lac Magera | NEG |
| AM192 | 42,395 | *Phrynobatrachus versicolor* | G. De Witte | 12-27 Sept 1934 | DRC | Col between Sabinyo and Gahinga - 2600m | Virunga | Kundhuru ya tohuve | NEG |
| AM193 | 42,394 | *Phrynobatrachus versicolor* | G. De Witte | 12-27 Sept 1934 | DRC | Col between Sabinyo and Gahinga - 2600m | Virunga | Kundhuru ya tohuve | NEG |
| AM194 | 42,393 | *Phrynobatrachus versicolor* | G. De Witte | 12-27 Sept 1934 | DRC | Col between Sabinyo and Gahinga - 2600m | Virunga | Kundhuru ya tohuve | NEG |
| AM195 | 42,392 | *Phrynobatrachus versicolor* | G. De Witte | 12-27 Sept 1934 | DRC | Col between Sabinyo and Gahinga - 2600m | Virunga | Kundhuru ya tohuve | NEG |
| AM196 | 42,391 | *Phrynobatrachus versicolor* | G. De Witte | 12-27 Sept 1934 | DRC | Col between Sabinyo and Gahinga - 2600m | Virunga | Kundhuru ya tohuve | NEG |
| AM197 | 42,390 | *Phrynobatrachus versicolor* | G. De Witte | 12-27 Sept 1934 | DRC | Col between Sabinyo and Gahinga - 2600m | Virunga | Kundhuru ya tohuve | NEG |
| AM198 | 42,389 | *Phrynobatrachus versicolor* | G. De Witte | 12-27 Sept 1934 | DRC | Col between Sabinyo and Gahinga - 2600m | Virunga | Kundhuru ya tohuve | NEG |
| AM199 | 42,388 | *Phrynobatrachus versicolor* | G. De Witte | 12-27 Sept 1934 | DRC | Col between Sabinyo and Gahinga - 2600m | Virunga | Kundhuru ya tohuve | NEG |
| AM200 | 42,387 | *Phrynobatrachus versicolor* | G. De Witte | 12-27 Sept 1934 | DRC | Col between Sabinyo and Gahinga - 2600m | Virunga | Kundhuru ya tohuve | NEG |
| AM201 | 42,386 | *Phrynobatrachus versicolor* | G. De Witte | 12-27 Sept 1934 | DRC | Col between Sabinyo and Gahinga - 2600m | Virunga | Kundhuru ya tohuve | NEG |
| AM202 | 55428 | *Hyperolius castaneus* | G. De Witte | 27 Jun - 12 Jul 1934 | DRC | Nyakibumba - 2000m | Virunga | Marais de Kikese | NEG |
| AM203 | 55427 | *Hyperolius castaneus* | G. De Witte | 27 Jun - 12 Jul 1934 | DRC | Nyakibumba - 2000m | Virunga | Marais de Kikese | NEG |
| AM204 | 55426 | *Hyperolius castaneus* | G. De Witte | 27 Jun - 12 Jul 1934 | DRC | Nyakibumba - 2000m | Virunga | Marais de Kikese | NEG |
| AM205 | 55425 | *Hyperolius castaneus* | G. De Witte | 27 Jun - 12 Jul 1934 | DRC | Nyakibumba - 2000m | Virunga | Marais de Kikese | NEG |
| AM206 | 55424 | *Hyperolius castaneus* | G. De Witte | 27 Jun - 12 Jul 1934 | DRC | Nyakibumba - 2000m | Virunga | Marais de Kikese | NEG |
| AM207 | 55423 | *Hyperolius castaneus* | G. De Witte | 27 Jun - 12 Jul 1934 | DRC | Nyakibumba - 2000m | Virunga | Marais de Kikese | NEG |
| AM208 | 55422 | *Hyperolius castaneus* | G. De Witte | 27 Jun - 12 Jul 1934 | DRC | Nyakibumba - 2000m | Virunga | Marais de Kikese | NEG |
| AM209 | 55421 | *Hyperolius castaneus* | G. De Witte | 27 Jun - 12 Jul 1934 | DRC | Nyakibumba - 2000m | Virunga | Marais de Kikese | NEG |

**H. constellatus* used to be a subspecies of *H. castaneus* and Laurent gave the latter name to these specimens. It has now been given separate species status based on genetics, morphology and allopatric distribution [46].
