# Supplementary material for: Assessing the Threat of Amphibian Chytrid Fungus in the Albertine Rift: Past, Present and Future
Source: PLoS One. 2015 Dec 28;10(12):e0145841. doi: 10.1371/journal.pone.0145841 (PMC4692535; doi:10.1371/journal.pone.0145841)
Supplement: S2 Table — Elevations are in meters above sea level. (DOCX) [file pone.0145841.s005.docx]

|  | **ID** | **Scientific Name (Genus)** | **Scientific Name (Species if known)** | **Collector** | **Date of Collection** | **Country** | **Elevation** | **Preservation Method** | **Field Site** | **PCR results** |
| --- | --- | --- | --- | --- | --- | --- | --- | --- | --- | --- |
| 1 | None | *Ptychadena* |  | Wilbur Lukwago | 6/1/2013 | Uganda | 1076 | Ethanol | Budongo Forest | NEG |
| 2 | None | *Ptychadena* |  | Wilbur Lukwago | 6/1/2013 | Uganda | 1040 | Ethanol | Budongo Forest | NEG |
| 3 | None | *Ptychadena* |  | Wilbur Lukwago | 6/1/2013 | Uganda | 1081 | Ethanol | Budongo Forest | NEG |
| 4 | None | *Leptopelis* |  | Wilbur Lukwago | 6/1/2013 | Uganda | 1033 | Ethanol | Budongo Forest | NEG |
| 5 | None | *Phrynobatrachus* |  | Wilbur Lukwago | 6/1/2013 | Uganda | 1098 | Ethanol | Budongo Forest | NEG |
| 6 | None | *Phrynobatrachus* |  | Wilbur Lukwago | 6/1/2013 | Uganda | 1057 | Ethanol | Budongo Forest | NEG |
| 7 | None | *Ptychadena* |  | Wilbur Lukwago | 6/1/2013 | Uganda | 1042 | Ethanol | Budongo Forest | NEG |
| 8 | None | *Arthroleptis* |  | Wilbur Lukwago | 6/1/2013 | Uganda | 1032 | Ethanol | Budongo Forest | **POS** |
| 9 | None | *Phrynobatrachus* |  | Wilbur Lukwago | 6/1/2013 | Uganda | 1047 | Ethanol | Budongo Forest | **POS** |
| 10 | None | *Xenopus* |  | Wilbur Lukwago | 6/1/2013 | Uganda | 1023 | Ethanol | Budongo Forest | NEG |
| 11 | None | *Hyperolius* |  | Wilbur Lukwago | 6/1/2013 | Uganda | 1076 | Ethanol | Budongo Forest | **POS** |
| 12 | None | *Ptychadena* |  | Wilbur Lukwago | 6/1/2013 | Uganda | 1077 | Ethanol | Budongo Forest | NEG |
| 13 | None | *Ptychadena* |  | Wilbur Lukwago | 6/1/2013 | Uganda | 1076 | Ethanol | Budongo Forest | NEG |
| 14 | None | *Amietophryus* |  | Wilbur Lukwago | 6/1/2013 | Uganda | 1071 | Ethanol | Budongo Forest | NEG |
| 15 | None | *Ptychadena* |  | Wilbur Lukwago | 6/1/2013 | Uganda | 1077 | Ethanol | Budongo Forest | NEG |
| 16 | None | *Xenopus* |  | Wilbur Lukwago | 6/1/2013 | Uganda | 1040 | Ethanol | Budongo Forest | NEG |
| 17 | None | *Ptychadena* |  | Wilbur Lukwago | 6/1/2013 | Uganda | 1077 | Ethanol | Budongo Forest | NEG |
| 18 | None | *Ptychadena* |  | Wilbur Lukwago | 6/1/2013 | Uganda | 1053 | Ethanol | Budongo Forest | NEG |
| 19 | None | *Hoplobatrachus* | *occipitalis* | Wilbur Lukwago | 6/1/2013 | Uganda | 1033 | Ethanol | Budongo Forest | NEG |
| 20 | None | *Amietophrynus* |  | Wilbur Lukwago | 6/1/2013 | Uganda | 1114 | Ethanol | Budongo Forest | NEG |
| 21 | None | *Ptychadena* |  | DF Oren | 4/1/1965 | Uganda |  | Ethanol | Budongo Forest | NEG |
| 22 | None | *Ptychadena* |  | DF Oren | 4/1/1965 | Uganda |  | Ethanol | Budongo Forest | NEG |
| 23 | None | *Ptychadena* |  | DF Oren | 4/1/1965 | Uganda |  | Ethanol | Budongo Forest | NEG |
| 24 | None | *Ptychadena* |  | DF Oren | 4/1/1965 | Uganda |  | Ethanol | Budongo Forest | NEG |
| 25 | None | *Ptychadena* |  | DF Oren | 4/1/1965 | Uganda |  | Ethanol | Budongo Forest | NEG |
| 26 | NUZN046 | *Hylarana* | *galamensis* | JB Goodman | 7/1/1992 | Uganda |  | Ethanol | Itwara Forest | NEG |
| 27 | NUZN044 | *Hylarana* | *galamensis* | JB Goodman | 7/1/1992 | Uganda |  | Ethanol | Itwara Forest | NEG |
| 28 | None | *Rana* | *angolensis* | M. Behangana | 2/1/1994 | Uganda |  | Formalin | Semuliki Forest | NEG |
| 29 | None | *Bufo* |  | M. Behangana | 2/1/1994 | Uganda |  | Formalin | Semuliki Forest | INDETERMINATE |
| 30 | NUZN041 | *Amietophrynus* | *regularis* | JB Goodman | 7/1/1992 | Uganda |  | Formalin | Itwara Forest | NEG |
| 31 | NUZN042 | *Amietophrynus* | *regularis* | JB Goodman | 7/1/1992 | Uganda |  | Formalin | Itwara Forest | NEG |
| 32 |  | *Bufo* |  |  | 4/1/1971 | Uganda |  | Formalin | Budongo Forest | NEG |
| 33 |  | *Bufo* |  |  | 4/1/1971 | Uganda |  | Formalin | Budongo Forest | NEG |
| 34 |  | *Bufo* |  |  | 4/1/1971 | Uganda |  | Formalin | Budongo Forest | NEG |
| 35 |  | *Bufo* |  |  | 4/1/1971 | Uganda |  | Formalin | Budongo Forest | INDETERMINATE |
| 36 | Wahirindi pad | *Ptychadena* |  | M. Behangana | 4/25/2010 | Uganda |  | Ethanol | Kabwoya? | INDETERMINATE |
| 37 |  | *Hyperolius* | *cinnamomeoventris* | M. Behangana | 8/1/2010 | Uganda |  | Ethanol | Kabwoya -R. Hohwa | NEG |
| 38 |  | *Ptychadena* | *nilotica* | M. Behangana | 8/1/2010 | Uganda |  | Ethanol | Kabwoya -R. Hohwa - bridge | NEG |
| 39 | Kaso Pad3 | *Ptychadena* | *porosissima* | M. Behangana | 5/24/2010 | Uganda |  | Ethanol | Kabwoya? | NEG |
| 40 | R.hohwa | *Ptychadena* | *chrysogaster* | M. Behangana | 8/1/2010 | Uganda |  | Ethanol | Kabwoya | NEG |
| 41 | R. Weiga bridge | *Ptychadena* |  | M. Behangana | 5/1/2010 | Uganda |  | Ethanol | Kabwoya | INDETERMINATE |
| 42 | Kazinga Channel | *Ptychadena* |  | JB Goodman | 2/1/1970 | Uganda |  | Formalin | Queen Elizabeth | INDETERMINATE |
| 43 | Kazinga Channel | *Ptychadena* |  | JB Goodman | 2/1/1970 | Uganda |  | Formalin | Queen Elizabeth | INDETERMINATE |
| 44 | Kazinga Channel | *Ptychadena* |  | JB Goodman | 2/1/1970 | Uganda |  | Formalin | Queen Elizabeth | INDETERMINATE |
| 45 | Kazinga Channel | *Ptychadena* |  | JB Goodman | 2/1/1970 | Uganda |  | Formalin | Queen Elizabeth | INDETERMINATE |
| 46 | Kazinga Channel | *Ptychadena* |  | JB Goodman | 2/1/1970 | Uganda |  | Formalin | Queen Elizabeth | INDETERMINATE |
| 47 | Kazinga Channel | *Ptychadena* |  | JB Goodman | 2/1/1970 | Uganda |  | Formalin | Queen Elizabeth | INDETERMINATE |
| 48 | Kazinga Channel | *Ptychadena* |  | JB Goodman | 2/1/1970 | Uganda |  | Formalin | Queen Elizabeth | INDETERMINATE |
| 49 | Kazinga Channel | *Ptychadena* |  | JB Goodman | 2/1/1970 | Uganda |  | Formalin | Queen Elizabeth | NEG |
