# Supplementary material for: Assessing the Threat of Amphibian Chytrid Fungus in the Albertine Rift: Past, Present and Future
Source: PLoS One. 2015 Dec 28;10(12):e0145841. doi: 10.1371/journal.pone.0145841 (PMC4692535; doi:10.1371/journal.pone.0145841)
Supplement: S3 Table — Elevations are in meters above sea level. (DOCX) [file pone.0145841.s006.docx]

| **ID** | ***Genus and Species if known*** | **Date of Collection** | **Country** | **Elevation** | **Northings** | **Eastings** | **Field Site** |
| --- | --- | --- | --- | --- | --- | --- | --- |
| 6836 | *Hyperolius castaneus* | 11/2011 | DRC | 2312 | -2.3251 | 28.72902 | Kahuzi-Biega NP, Bugulumiza |
| 6837 | *Afrixalus cf. laevis* | 11/2011 | DRC | 2312 | -2.3251 | 28.72902 | Kahuzi-Biega NP, Bugulumiza |
| 6838 | *Leptopelis kivuensis* | 11/2011 | DRC | 2312 | -2.3251 | 28.72902 | Kahuzi-Biega NP, Bugulumiza |
| 6839 | *Leptopelis kivuensis* | 11/2011 | DRC | 2312 | -2.3251 | 28.72902 | Kahuzi-Biega NP, Bugulumiza |
| 6840 | *Afrixalus cf. laevis* | 11/2011 | DRC | 2312 | -2.3251 | 28.72902 | Kahuzi-Biega NP, Bugulumiza |
| 6844 | *Hyperolius castaneus* | 11/2011 | DRC | 2312 | -2.3251 | 28.72902 | Kahuzi-Biega NP, Bugulumiza |
| 6845 | *Hyperolius castaneus* | 11/2011 | DRC | 2312 | -2.3251 | 28.72902 | Kahuzi-Biega NP, Bugulumiza |
| 6849 | *Hyperolius discodactylus* | 11/2011 | DRC | 2312 | -2.3251 | 28.72902 | Kahuzi-Biega NP, Bugulumiza |
| 6851 | *Afrixalus cf.laevis* | 11/2011 | DRC | 2312 | -2.3251 | 28.72902 | Kahuzi-Biega NP, Bugulumiza |
| 6853 | *Hyperolius castaneus* | 11/2011 | DRC | 2312 | -2.3251 | 28.72902 | Kahuzi-Biega NP, Bugulumiza |
| 6855 | *Hyperolius castaneus* | 11/2011 | DRC | 2312 | -2.3251 | 28.72902 | Kahuzi-Biega NP, Bugulumiza |
| 6858 | *Afrixalus cf. laevis* | 11/2011 | DRC | 2312 | -2.3251 | 28.72902 | Kahuzi-Biega NP, Bugulumiza |
| 6868 | *Leptopelis karissimbensis* | 11/2011 | DRC | 1930 | -2.30926 | 28.64935 | Kahuzi-Biega NP, Madiriri |
| 6870 | *Leptopelis karissimbensis* | 11/2011 | DRC | 1930 | -2.30926 | 28.64935 | Kahuzi-Biega NP, Madiriri |
| 6871 | *Leptopelis cf. karissimbensis* | 11/2011 | DRC | 1930 | -2.30926 | 28.64935 | Kahuzi-Biega NP, Madiriri |
| 6872 | *Leptopelis karissimbensis* | 11/2011 | DRC | 1930 | -2.30926 | 28.64935 | Kahuzi-Biega NP, Madiriri |
| 6874 | *Xenopus wittei* | 11/2011 | DRC | 1930 | -2.30926 | 28.64935 | Kahuzi-Biega NP, Madiriri |
| 6875 | *Xenopus sp* | 11/2011 | DRC | 1930 | -2.30926 | 28.64935 | Kahuzi-Biega NP, Madiriri |
| 6877 | *Xenopus wittei* | 11/2011 | DRC | 1930 | -2.30926 | 28.64935 | Kahuzi-Biega NP, Madiriri |
| 6883 | *Leptopelis karissimbensis* | 11/2011 | DRC | 2311 | -2.27359 | 28.66297 | Kahuzi-Biega NP, Mugaba |
| 6884 | *Leptopelis sp* | 11/2011 | DRC | 2311 | -2.27359 | 28.66297 | Kahuzi-Biega NP, Mugaba |
| 6885 | *Leptopelis sp* | 11/2011 | DRC | 2311 | -2.27359 | 28.66297 | Kahuzi-Biega NP, Mugaba |
| 6886 | *Leptopelis karissimbensis* | 11/2011 | DRC | 2311 | -2.27359 | 28.66297 | Kahuzi-Biega NP, Mugaba |
| 6887 | *Leptopelis karissimbensis* | 11/2011 | DRC | 2311 | -2.27359 | 28.66297 | Kahuzi-Biega NP, Mugaba |
| 6888 | *Hyperolius castaneus* | 11/2011 | DRC | 2311 | -2.27359 | 28.66297 | Kahuzi-Biega NP, Mugaba |
| 6889 | *Hyperolius castaneus* | 11/2011 | DRC | 2311 | -2.27359 | 28.66297 | Kahuzi-Biega NP, Mugaba |
| 6890 | *Leptopelis kivuensis* | 11/2011 | DRC | 2311 | -2.27359 | 28.66297 | Kahuzi-Biega NP, Mugaba |
| 6891 | *Leptopelis kivuensis* | 11/2011 | DRC | 2311 | -2.27359 | 28.66297 | Kahuzi-Biega NP, Mugaba |
| 7830 | *Afrixalus sp* | 3/22/13 | DRC | 1151 | -3.519 | 28.419 | Itombwe Massif - Mwana |
| 7836 | *Hyperolius sp* | 3/22/13 | DRC | 1151 | -3.519 | 28.419 | Itombwe Massif - Mwana |
| 7839 | *Hyperolius sp* | 3/22/13 | DRC | 1151 | -3.519 | 28.419 | Itombwe Massif - Mwana |
| 7842 | *Leptopelis sp* | 3/22/13 | DRC | 1145 | -3.52 | 28.419 | Itombwe Massif - Mwana |
| 7843 | *Leptopelis sp* | 3/22/13 | DRC | 1145 | -3.52 | 28.419 | Itombwe Massif - Mwana |
| 7845 | *Leptopelis kivuensis* | 3/23/13 | DRC | 1145 | -3.52 | 28.419 | Itombwe Massif - Mwana |
| 7861 | *Hyperiolus sp* | 3/25/13 | DRC | 1261 | -3.296 | 28.331 | Itombwe Massif - Mwana |
| 7862 | *Hyperiolus sp* | 3/25/13 | DRC | 1261 | -3.296 | 28.331 | Itombwe Massif - Mwana |
| 7863 | *Hyperiolus sp* | 3/25/13 | DRC | 1261 | -3.296 | 28.331 | Itombwe Massif - Mwana |
| 7865 | *Afrixalus sp* | 3/25/13 | DRC | 1261 | -3.296 | 28.331 | Itombwe Massif - Mwana |
| 7883 | *Afrixalus osorioi* | 12/15/13 | DRC | 1056 | -1.782 | 27.533 | Kahuzi-Biega NP, Kasese |
| 7886 | *Phrynobatrachus sp* | 12/17/13 | DRC | 1095 | -1.844 | 27.608 | Kahuzi-Biega NP, Kasese |
| 7892 | *Hyperolius sp* | 12/26/13 | DRC | 1128 | -1.757 | 27.449 | Kahuzi-Biega NP, Kasese |
| 7933 | *Afrixalus osorioi* | 9/3/13 | DRC | 664 | -2.558 | 27.994 | Lowland Kahuzi Biega,Nzovu |
| 7936 | *Hyperolius sp* | 9/3/13 | DRC | 664 | -2.558 | 27.994 | Lowland Kahuzi Biega,Nzovu |
| 7937 | *Afrixalus sp* | 9/3/13 | DRC | 664 | -2.558 | 27.994 | Lowland Kahuzi Biega,Nzovu |
| 7938 | *Afrixalus sp* | 9/3/13 | DRC | 664 | -2.558 | 27.994 | Lowland Kahuzi Biega,Nzovu |
| 7949 | *Leptopelis kivuensis* | 9/6/13 | DRC | 1255 | -2.589 | 28.051 | Lowland Kahuzi Biega,Nzovu |
| 7953 | *Arthroleptus sp* | 9/6/13 | DRC | 837 | -2.605 | 28.074 | Lowland Kahuzi Biega,Nzovu |
| 7996 | *Phrynobatrachus kreffti* | 9/27/13 | DRC | 1297 | -2.35 | 28.081 | Lowland Kahuzi Biega,Nzovu |
| 9732 | *Ptychadena chrysogaster* | 5/13/12 | DRC |  | -3.8756 | 27.92523 | Itombwe Massif, Mukoloka |
| 9733 | *Ptychadena sp1* | 5/13/12 | DRC |  | -3.8756 | 27.92523 | Itombwe Massif, Mukoloka |
| 9734 | *Afrixalus quadrivittatus* | 5/13/12 | DRC |  | -3.8756 | 27.92523 | Itombwe Massif, Mukoloka |
| 9738 | *Phlyctimantis verrucosus* | 5/15/12 | DRC |  | -3.90178 | 28.01918 | Itombwe Massif, Bilolo |
| 9742 | *Hyperolius frontalis* | 5/16/12 | DRC |  | -3.87501 | 28.03029 | Itombwe Massif, Inawaloua |
| 9752 | *Hyperolius sp4* | 5/17/12 | DRC |  | -3.85572 | 28.04106 | Itombwe Massif, Simunabi |
| 9765 | *Ptychadena spA* | 5/28/12 | DRC |  | -5.064929 | 28.915692 | Kobobo and Luama Katanga Reserve, Bandera |
| 9770 | *Leptopelis kivuensis* | 5/28/12 | DRC |  | -5.064943 | 28.920255 | Kobobo and Luama Katanga Reserve, Bandera |
| 9774 | *Afrixalus sp1* | 8/9/12 | DRC |  | -3.14474 | 28.99859 | Itombwe Massif, Mulenge |
| 9782 | *Afrixalus sp3* | 8/9/12 | DRC |  | -3.14474 | 28.99859 | Itombwe Massif, Mulenge |
| 9784 | *Hyperolius sp2* | 8/9/12 | DRC |  | -3.14474 | 28.99859 | Itombwe Massif, Mulenge |
| 9790 | *Hyperolius sp5* | 8/9/12 | DRC |  | -3.19796 | 28.99244 | Itombwe Massif, Mulenge |
| 9792 | *Hyperolius sp7* | 8/9/2012 | DRC |  | -3.19796 | 28.99244 | Itombwe Massif, Mulenge |
| 9830 | *Phrynobatrachus sp1* | 11/1/12 | DRC | 1281 | -5.258 | 28.888 | Kobobo and Luama Katanga Reserve, |
| 9847 | *Hyperolius sp1* | 11/5/12 | DRC | 1962 | -5.24 | 29.105 | Kobobo and Luama Katanga Reserve, |
| 9855 | *Arthroleptis sp1* | 11/14/12 | DRC | 1725 | -5.164 | 29.113 | Kobobo and Luama Katanga Reserve, |
| 9875 | *Afrixalus sp* | 8/31/13 | DRC | 686 | -1.384 | 27.198 | Punia Gorilla Reserve, west of Kahuzi Biega |
| 9881 | *Afrixalus sp* | 9/12/13 | DRC | 588 | -1.637 | 27.115 | Punia Gorilla Reserve, west of Kahuzi Biega |
| 9883 | *Afrixalus wittei* | 9/15/13 | DRC | 588 | -1.637 | 27.115 | Punia Gorilla Reserve, west of Kahuzi Biega |
| 9890 | *Afrixalus wittei* | 3/18/13 | DRC | 1128 | -3.44 | 28.351 | Itombwe Massif, Mwana |
| 9891 | *Afrixalus wittei* | 3/18/13 | DRC | 1128 | -3.44 | 28.351 | Itombwe Massif, Mwana |
| 9895 | *Hyperolius sp* | 3/18/13 | DRC | 1128 | -3.44 | 28.351 | Itombwe Massif, Mwana |
| 9896 | *Hyperolius robustus* | 3/18/13 | DRC | 1128 | -3.44 | 28.351 | Itombwe Massif, Mwana |
| 9897 | *Amietophrynus sp* | 3/18/13 | DRC | 1128 | -3.44 | 28.351 | Itombwe Massif, Mwana |
| 9899 | *Hyperolius sp* | 3/18/13 | DRC | 1128 | -3.44 | 28.351 | Itombwe Massif, Mwana |
| 9904 | *Hyperolius sp* | 3/18/13 | DRC | 1128 | -3.44 | 28.351 | Itombwe Massif, Mwana |
| 9905 | *Hyperolius sp* | 3/18/13 | DRC | 1128 | -3.44 | 28.351 | Itombwe Massif, Mwana |
| 9910 | *Hyperolius sp* | 3/18/13 | DRC | 1128 | -3.44 | 28.351 | Itombwe Massif, Mwana |
| 9918 | *Afrixalus sp* | 3/18/13 | DRC | 1130 | -3.445 | 28.356 | Itombwe Massif, Mwana |
| 9921 | *Hyperolius sp* | 3/18/13 | DRC | 1130 | -3.445 | 28.356 | Itombwe Massif, Mwana |
| 9963 | *Phrynobatrachus sp1* | 11/13/12 | DRC | 1032 | -5.08 | 28.734 | Luama-Katanga Reserve |
| 9964 | *Phrynobatrachus sp1* | 11/13/12 | DRC | 1032 | -5.08 | 28.734 | Luama-Katanga Reserve |
| 9972 | *Ptychadena sp1* | 11/15/12 | DRC | 865 | -5.152 | 28.744 | Luama-Katanga Reserve |
| 9973 | *Hyperolius sp1* | 11/15/12 | DRC | 865 | -5.152 | 28.744 | Luama-Katanga Reserve |
| 9979 | *Ptychadena sp1* | 11/15/12 | DRC | 858 | -5.149 | 28.742 | Luama-Katanga Reserve |
| 10001 | *Afrixalus osorioi* | 12/5/13 | DRC | 702 | -1.674 | 28.128 | Kahuzi-Biega NP, Itebero |
| 10002 | *Afrixalus osorioi* | 12/5/13 | DRC | 702 | -1.674 | 28.128 | Kahuzi-Biega NP, Itebero |
| 10038 | *Leptopelis sp* | 12/25/13 | DRC | 1002 | -1.925 | 28.019 | Kahuzi-Biega NP, Itebero |
| 10489 | *Hyperolius castaneus* | 12/11/13 | DRC | 2019 | -3.14535 | 28.99899 | Itombwe Massif, Mulenge |
| 10493 | *Hyperolius castaneus* | 12/12/13 | DRC | 2037 | -3.14594 | 29.00045 | Itombwe Massif, Mulenge |
| 10494 | *Leptopelis sp* | 12/8/13 | DRC | 2053 | -3.15388 | 28.9937 | Itombwe Massif, Mulenge |
| 10498 | *Hyperolius castaneus* | 12/10/13 | DRC | 2065 | -3.14857 | 28.99335 | Itombwe Massif, Mulenge |
| 10499 | *Hyperolius castaneus* | 12/12/13 | DRC | 2037 | -3.14594 | 29.00045 | Itombwe Massif, Mulenge |
| 10501 | *Hyperolius constellatus* | 12/10/13 | DRC | 2065 | -3.14857 | 28.99335 | Itombwe Massif, Mulenge |
| 10502 | *Hyperolius castaneus* | 12/12/13 | DRC | 2037 | -3.14594 | 29.00045 | Itombwe Massif, Mulenge |
| 10508 | *Hyperolius castaneus* | 12/12/13 | DRC | 2037 | -3.14594 | 29.00045 | Itombwe Massif, Mulenge |
| 10510 | *Hyperolius sp* | 12/13/13 | DRC | 2054 | -3.13772 | 28.9919 | Itombwe Massif, Mulenge |
| 10514 | *Hyperolius sp* | 12/11/13 | DRC | 2019 | -3.14535 | 28.99899 | Itombwe Massif, Mulenge |
| 10516 | *Hyperolius nasutus* | 12/13/13 | DRC | 1991 | -3.13517 | 28.99697 | Itombwe Massif, Mulenge |
| 10519 | *Leptopelis sp* | 12/8/13 | DRC | 2053 | -3.15388 | 28.9937 | Itombwe Massif, Mulenge |
| 10520 | *Hyperolius sp* | 12/13/13 | DRC | 1991 | -3.13517 | 28.99697 | Itombwe Massif, Mulenge |
| 10521 | *Hyperolius sp* | 12/11/13 | DRC | 2019 | -3.14535 | 28.99899 | Itombwe Massif, Mulenge |
| 10523 | *Hyperolius sp* | 12/13/13 | DRC | 1991 | -3.13517 | 28.99697 | Itombwe Massif, Mulenge |
| 10524 | *Xenopus sp* | 12/8/13 | DRC | 2046 | -3.15285 | 28.992 | Itombwe Massif, Mulenge |
| 10525 | *Hyperolius sp* | 12/13/13 | DRC | 1991 | -3.13517 | 28.99697 | Itombwe Massif, Mulenge |
| 10527 | *Arthroleptis sp* | 12/12/13 | DRC | 2091 | -3.14551 | 28.99653 | Itombwe Massif, Mulenge |
| 10529 | *Hyperolius sp* | 12/8/13 | DRC | 2046 | -3.15285 | 28.992 | Itombwe Massif, Mulenge |
| 10532 | *Amietia sp* | 12/10/13 | DRC | 2061 | -3.14587 | 28.99501 | Itombwe Massif, Mulenge |
| 10544 | *Hyperolius sp* | 12/13/13 | DRC | 2054 | -3.13772 | 28.9919 | Itombwe Massif, Mulenge |
| 10545 | *Hyperolius castaneus* | 12/8/13 | DRC | 2046 | -3.15285 | 28.992 | Itombwe Massif, Mulenge |
| 29431 | *Leptopelis kivuensis* | 11/2011 | DRC | 2311 | -2.27359 | 28.66297 | Kahuzi-Biega NP, Mugaba |
| 29434 | *Afrixalus cf. laevis* | 11/2011 | DRC | 2311 | -2.27359 | 28.66297 | Kahuzi-Biega NP, Mugaba |
| 29435 | *Amietia sp* | 11/2011 | DRC | 2311 | -2.27359 | 28.66297 | Kahuzi-Biega NP, Mugaba |
| 108878 | *Phrynobatrachus asper* | 2-8 Aug 1950 | DRC | 2450-2500m |  |  | Itombwe Massif, River Makenda |
| MUSE 10060 | *Phrynobatrachus sp* | 2/25/14 | DRC | 2208 | -3.817 | 28.969 | North Balala Forest, Ndobo |
| MUSE 10062 | *Phrynobatrachus sp* | 2/25/14 | DRC | 2208 | -3.817 | 28.969 | North Balala Forest, Ndobo |
| MUSE 10063 | *Phrynobatrachus sp* | 2/25/14 | DRC | 2208 | -3.817 | 28.969 | North Balala Forest, Ndobo |
| MUSE 10064 | *Phrynobatrachus sp* | 2/25/14 | DRC | 2208 | -3.817 | 28.969 | North Balala Forest, Ndobo |
| MUSE 10068 | *Phrynobatrachus sp* | 2/25/14 | DRC | 2170 | -3.818 | 28.969 | North Balala Forest, Ndobo |
| MUSE 10102 | *Arthroleptis sp* | 3/3/14 | DRC | 2369 | -3.723 | 28.943 | North Balala Forest, Namulombwa |
| MUSE 10119 | *Leptopelis sp* | 4/10/14 | DRC | 1886 | -3.22 | 28.656 | Itombwe Massif, Kakanga |
| MUSE 10124 | *Leptopelis sp* | 4/13/14 | DRC | 1528 | -3.383 | 28.567 | Itombwe Massif, Kasenge |
| MUSE 10129 | *Phrynobatrachus sp* | 4/16/14 | DRC | 1879 | -3.253 | 28.543 | Itombwe Massif, Yambanga |
| MUSE 10130 | *Phrynobatrachus sp* | 4/16/14 | DRC | 2024 | -3.234 | 28.519 | Itombwe Massif, Misebu |
| 4 | *Hyperolius castaneus* | 3/5/11 | Rwanda | 1980 | -2.48100177 | 29.1549139 | Nyungwe NP,  Kamiranzovu |
| 15 | *Hyperolius castaneus* | 3/5/11 | Rwanda | 1980 | -2.48100177 | 29.1549139 | Nyungwe NP,  Kamiranzovu |
| 17 | *Hyperolius castaneus* | 3/5/11 | Rwanda | 1980 | -2.48100177 | 29.1549139 | Nyungwe NP,  Kamiranzovu |
| 18 | *Hyperolius castaneus* | 3/5/11 | Rwanda | 1980 | -2.48100177 | 29.1549139 | Nyungwe NP,  Kamiranzovu |
| 22 | *Hyperolius castaneus* | 3/5/11 | Rwanda | 1980 | -2.48100177 | 29.1549139 | Nyungwe NP,  Kamiranzovu |
| 27 | *Hyperolius castaneus* | 3/5/11 | Rwanda | 1980 | -2.48100177 | 29.1549139 | Nyungwe NP,  Kamiranzovu |
| 30 | *Hyperolius castaneus* | 3/5/11 | Rwanda | 1980 | -2.48100177 | 29.1549139 | Nyungwe NP,  Kamiranzovu |
| 31 | *Hyperolius castaneus* | 3/5/11 | Rwanda | 1980 | -2.48100177 | 29.1549139 | Nyungwe NP,  Kamiranzovu |
| 36 | *Hyperolius castaneus* | 3/5/11 | Rwanda | 1980 | -2.48100177 | 29.1549139 | Nyungwe NP,  Kamiranzovu |
| 37 | *Hyperolius castaneus* | 3/5/11 | Rwanda | 1980 | -2.48100177 | 29.1549139 | Nyungwe NP,  Kamiranzovu |
| 38 | *Hyperolius castaneus* | 3/5/11 | Rwanda | 1980 | -2.48100177 | 29.1549139 | Nyungwe NP,  Kamiranzovu |
| 42 | *Amietia sp* | 3/6/11 | Rwanda | 1980 | -2.48396 | 29.15307 | Nyungwe NP,  Kamiranzovu |
| 44 | *Amietia sp* | 3/6/11 | Rwanda | 1980 | -2.48396 | 29.15307 | Nyungwe NP,  Kamiranzovu |
| 52 | *Afrixalus cf. laevis* | 3/8/11 | Rwanda | 1980 | -2.4772206 | 29.1581407 | Nyungwe NP,  Kamiranzovu |
| 53 | *Afrixalus cf. laevis* | 3/8/11 | Rwanda | 1980 | -2.4772206 | 29.1581407 | Nyungwe NP,  Kamiranzovu |
| 63 | *Arthroleptis sp* | 3/8/11 | Rwanda | 1980 | -2.4772206 | 29.1581407 | Nyungwe NP,  Kamiranzovu |
| 64 | *Arthroleptis sp* | 3/8/11 | Rwanda | 1980 | -2.4772206 | 29.1581407 | Nyungwe NP,  Kamiranzovu |
| BS-3 | *Hyperolius discodactylus* | 12/5/10 | Rwanda | 2652 | -2.45556529 | 29.2492775 | Nyungwe NP,  Bigugu |
| BS-6 | *Hyperolius discodactylus* | 12/5/10 | Rwanda | 2652 | -2.45556529 | 29.2492775 | Nyungwe NP,  Bigugu |
| BS-7 | *Hyperolius discodactylus* | 12/5/10 | Rwanda | 2652 | -2.45556529 | 29.2492775 | Nyungwe NP,  Bigugu |
| BS-9A | *Hyperolius discodactylus* | 12/5/10 | Rwanda | 2652 | -2.45556529 | 29.2492775 | Nyungwe NP,  Bigugu |
| DTR-12 | *Amietia sp.* | 12/3/10 | Rwanda | 1980 | -2.47680377 | 29.158523 | Nyungwe NP,  Kamiranzovu |
| DTR-20 | *Afrixalus cf. laevis* | 12/3/10 | Rwanda | 1980 | -2.47680377 | 29.158523 | Nyungwe NP,  Kamiranzovu |
| M2-4 | *Hyperolius castaneus* | 12/10/10 | Rwanda | 1915 | -2.54552289 | 28.9819364 | Nyungwe NP, Cyamudongo |
| M2-6 | *Hyperolius kivuensis* | 12/10/10 | Rwanda | 1915 | -2.54552289 | 28.9819364 | Nyungwe NP, Cyamudongo |
| M2-7 | *Hyperolius kivuensis* | 12/10/10 | Rwanda | 1915 | -2.54552289 | 28.9819364 | Nyungwe NP, Cyamudongo |
| M2-9 | *Hyperolius kivuensis* | 12/10/10 | Rwanda | 1915 | -2.54552289 | 28.9819364 | Nyungwe NP, Cyamudongo |
| OT11 | *Hyperolius castaneus* | 12/1/10 | Rwanda | 1980 | -2.481001 | 29.1549139 | Nyungwe NP,  Kamiranzovu |
| OT19 | *Hyperolius castaneus* | 12/1/10 | Rwanda | 1980 | -2.48100177 | 29.1549139 | Nyungwe NP,  Kamiranzovu |
| S1-5 | *Afrixalus cf. laevis* | 12/2/10 | Rwanda | 1980 | -2.4772206 | 29.1581407 | Nyungwe NP,  Kamiranzovu |
| ST1 | *Hyperolius castaneus* | 12/1/10 | Rwanda | 1980 | -2. 48457 | 29.15311 | Nyungwe NP,  Kamiranzovu |
| ST13 | *Hyperolius castaneus* | 12/1/10 | Rwanda | 1980 | -2. 48457 | 29.15311 | Nyungwe NP,  Kamiranzovu |
| ST21 | *Hyperolius castaneus* | 12/1/10 | Rwanda | 1980 | -2. 48457 | 29.15311 | Nyungwe NP,  Kamiranzovu |
| ST8 | *Hyperolius castaneus* | 12/1/10 | Rwanda | 1980 | -2. 48457 | 29.15311 | Nyungwe NP,  Kamiranzovu |
| V-15 | *Hyperolius kivuensis* | 12/10/10 | Rwanda | 1915 | -2.54529096 | 28.9850776 | Nyungwe NP, Cyamudongo |
| BIS 107 | *Leptopelis kivuensis* | 1/2011 | Uganda | 2185 | -1.044742 | 29.778215 | Bwindi Impenetrable NP,Ruhija |
| BIS 108 | *Leptopelis kivuensis* | 1/2011 | Uganda | 2185 | -1.044742 | 29.778215 | Bwindi Impenetrable NP,Ruhija |
| Bmj 210 | *Hyperolius viridiflavus pitmani* | 1/27/11 | Uganda | 1831 | -0.98684 | 29.635128 | Bwindi Impenetrable NP,Buhoma |
| Bmj 211 | *Leptopelis kivuensis* | 1/27/11 | Uganda | 1831 | -0.98684 | 29.635128 | Bwindi Impenetrable NP,Buhoma |
| Bmj 215 | *Xenopus cf wittei* | 1/27/11 | Uganda | 1831 | -0.98684 | 29.635128 | Bwindi Impenetrable NP,Buhoma |
| Bmj 216 | *Leptopelis sp* | 1/27/11 | Uganda | 1831 | -0.98684 | 29.635128 | Bwindi Impenetrable NP,Buhoma |
| Bmj 219 | *Amietia angolensis* | 1/27/11 | Uganda | 1831 | -0.98684 | 29.635128 | Bwindi Impenetrable NP,Buhoma |
| Bmj 225 | *Hyperolius viridiflavus pitmani* | 1/27/11 | Uganda | 1831 | -0.98684 | 29.635128 | Bwindi Impenetrable NP,Buhoma |
| Bmj 227 | *Hyperolius sp6* | 1/27/11 | Uganda | 1831 | -0.98684 | 29.635128 | Bwindi Impenetrable NP,Buhoma |
| Bmy 201 | *Leptopelis sp* | 1/25/11 | Uganda | 1479 | -0.991335 | 29.614636 | Bwindi Impenetrable NP,Buhoma |
| Bmy 205 | *Amietia sp* | 1/25/11 | Uganda | 1479 | -0.991335 | 29.614636 | Bwindi Impenetrable NP,Buhoma |
| Bmy 207 | *Hyperolius cinnamomeoventris* | 1/25/11 | Uganda | 1479 | -0.991335 | 29.614636 | Bwindi Impenetrable NP,Buhoma |
| Bmy 211 | *Hyperolius spx* | 1/25/11 | Uganda | 1479 | -0.991335 | 29.614636 | Bwindi Impenetrable NP,Buhoma |
| Bmy 212 | *Hyperolius spx* | 1/25/11 | Uganda | 1479 | -0.991335 | 29.614636 | Bwindi Impenetrable NP,Buhoma |
| Bny 219 | *Hyperolius spx* | 1/16/11 | Uganda | 2126 | -1.07859 | 29.746135 | Bwindi Impenetrable NP,Mubwindi |
| Bny 106 | *Leptopelis kivuensis* | 1/16/11 | Uganda | 2126 | -1.07859 | 29.746135 | Bwindi Impenetrable NP,Mubwindi |
| Ham 207 | *Amietia angolensis* | 1/13/11 | Uganda | 2134 | -1.034314 | 29.770891 | Bwindi Impenetrable NP,Ruhija |
| Kas 101 | *Leptopelis kivuensis* | 3/3/11 | Uganda | 2010 | -1.086344 | 29.752781 | Bwindi Impenetrable NP,Mubwindi |
| Kip 104 | *Hyperolius cinnamomeoventris* | 3/19/11 | Uganda | 1573 | -0.976046 | 29.686856 | Bwindi Impenetrable NP,Kitahurira |
| Kip 105 | *Hyperolius cinnamomeoventris* | 3/19/11 | Uganda | 1573 | -0.976046 | 29.686856 | Bwindi Impenetrable NP,Kitahurira |
| Kip 109 | *Hyperolius cinnamomeoventris* | 3/19/11 | Uganda | 1573 | -0.976046 | 29.686856 | Bwindi Impenetrable NP,Kitahurira |
| Kip 133 | *Hyperolius cinnamomeoventris* | 3/19/11 | Uganda | 1573 | -0.976046 | 29.686856 | Bwindi Impenetrable NP,Kitahurira |
| Kop 101 | *Ptychadena chrysogaster* | 3/19/11 | Uganda | 1438 | -0.977133 | 29.688101 | Bwindi Impenetrable NP,Kitahurira |
| Ksp 120 | *Hyperolius castaneus* | 3/3/11 | Uganda | 2076 | -1.088813 | 29.754191 | Bwindi Impenetrable NP,Mubwindi |
| Ksp 122 | *Leptopelis kivuensis* | 3/3/11 | Uganda | 2076 | -1.088813 | 29.754191 | Bwindi Impenetrable NP,Mubwindi |
| Mbw 105 | *Leptopelis kivuensis* | 2/26/11 | Uganda | 1963 | -1.019482 | 29.742124 | Bwindi Impenetrable NP,Mbwa swamp |
| Mbw 111 | *Leptopelis kivuensis* | 2/26/11 | Uganda | 1963 | -1.019482 | 29.742124 | Bwindi Impenetrable NP,Mbwa swamp |
| Mpr 101 | *Xenopus cf wittei* | 3/12/11 | Uganda | 2131 | -1.097593 | 29.699673 | Bwindi Impenetrable NP,Rushaga |
| Ndk 209 | *Ptychadena chrysogaster* | 1/21/11 | Uganda | 2151 | -1.099711 | 29.792364 | Bwindi Impenetrable NP,Ndego |
| NGO 101 | *Ptychadena achietae* | 1/2/11 | Uganda | 1418 | -0.895611 | 29.729942 | Bwindi Impenetrable NP,Ngoto |
| Nkf 102 | *Amietia angolensis* | 3/15/11 | Uganda | 1779 | -1.077609 | 29.644514 | Bwindi Impenetrable NP,Nkuringo |
| None | *Arthroleptis* | 6/1/13 | Uganda | 1032 | 1.724 | 31.528 | Budongo Forest |
| None | *Hyperolius* | 6/1/13 | Uganda | 1076 | 1.725 | 31.547 | Budongo Forest |
| None | *Phrynobatrachus* | 6/1/13 | Uganda | 1047 | 1.724 | 31.528 | Budongo Forest |
| Pd 101 | *Hyperolius castaneus* | 3/12/11 | Uganda | 2131 | -1.097593 | 29.699673 | Bwindi Impenetrable NP,Rushaga |
| Rsn 107 | *Hyperolius castaneus* | 3/9/11 | Uganda | 1870 | -1.119319 | 29.708218 | Bwindi Impenetrable NP,Rushaga |
| Rsr 101 | *Xenopus cf wittei* | 9/3/11 | Uganda | 1867 | -1.119693 | 29.705998 | Bwindi Impenetrable NP,Rushaga |
| Rw 302 | *Leptopelis kivuensis* | 1/13/11 | Uganda | 2138 | -1.050423 | 29.787267 | Bwindi Impenetrable NP,Ruhija |
| Rw 306 | *Leptopelis kivuensis* | 1/13/11 | Uganda | 2138 | -1.050423 | 29.787267 | Bwindi Impenetrable NP,Ruhija |
| Rw 311 | *Leptopelis kivuensis* | 1/13/11 | Uganda | 2138 | -1.050423 | 29.787267 | Bwindi Impenetrable NP,Ruhija |
