# Supplementary material for: Assessing the Threat of Amphibian Chytrid Fungus in the Albertine Rift: Past, Present and Future
Source: PLoS One. 2015 Dec 28;10(12):e0145841. doi: 10.1371/journal.pone.0145841 (PMC4692535; doi:10.1371/journal.pone.0145841)
Supplement: S4 Table — Elevations are in meters above sea level. (DOCX) [file pone.0145841.s007.docx]

| **Province** | **Dates** | **Elevation** | **Northings** | **Eastings** | **Locality** |
| --- | --- | --- | --- | --- | --- |
| Kabale | 1935 | 2006 | -1.281749 | 29.91509 | Lake Bunyonyi, Uganda |
| Fort Portal | 2006 | 1500 | 0.566548 | 30.3585889 | Kibale National Park, Uganda |
| West of Kivu | 1-Jan-07 | 820 | -1.8873059 | 28.4495 | Forest near Irangi, DRC |
| North Kivu | 12-Jul-08 | 841 | 0.66489 | 29.87811 | Kamango, DRC |
| Maniema | 9-Jun-09 | 794 | 4.67989 | 28.65341 | Lulimba, DRC |
| South Kivu | 23-Dec-09 | 1969–1973 | -2.22747 | 28.77922 | Tshibati, DRC |
| South Kivu | 21-Dec-09 | 1964–1979 | -2.2275 | 28.78017 | Nyakasanza, DRC |
| South Kivu | 24-Jun-08 | 1785–1816 | -3.37258 | 28.64308 | Kiandjo, Itombwe Plateau, DRC |
| North Kivu | 6–7 July 2008 | 2749–2767 | -0.12381 | 29.43167 | Mt. Tshiaberimu, Virunga National Park, DRC |
| South Kivu | 23–24 May 2009 | 2788–2843 | -3.07655 | 28.8104 | Komesha, Lake Lungwe, Itombwe Plateau, DRC |
| South Kivu | 21-Dec-09 | 1964–1979 | -2.2275 | 28.78017 | Nyakasanza, DRC |
| South Kivu | 11–12 Dec. 2010 | 1132 | -3.0288 | 28.28243 | Bizombo, DRC |
| South Kivu | 4 June 2008–5 June 2008 | 2262–2300 | -2.26344 | 28.66208 | Mugaba, Kahuzi-Biega National Park, DRC |
| South Kivu |  | 2262–2300 | -2.27288 | 28.66779 | Mugaba, Kahuzi-Biega National Park, DRC |
| South Kivu | 23–27 June 2008 | 1883–1965 | -3.35935 | 28.66581 | Miki, Itombwe Plateau, DRC |
| South Kivu |  | 1883–1965 | -3.37261 | 28.67794 | Miki, Itombwe Plateau, DRC |
| South Kivu | 23–24 May 2009 | 2788–2843 | -3.07655 | 28.8104 | Komesha, Lake Lungwe, Itombwe Plateau, DRC |
| South Kivu |  | 2788–2843 | -3.08847 | 28.81672 | Komesha, Lake Lungwe, Itombwe Plateau, DRC |
| South Kivu | 26-May-09 | 2450 | -3.00658 | 28.75005 | Kizuka, Itombwe Plateau, DRC |
| Katanga | 10 June 2009, 16 June 2009 | 970–974 | -5.05687 | 28.92151 | Force Bendera, DRC |
| Katanga |  | 970–974 | -5.06002 | 28.9236 | Force Bendera, DRC |
| South Kivu | 7-Jan-11 | 2848–2852 | -3.4314 | 29.01154 | Mitamba, DRC |
| Katanga |  | 2848–2852 | -3.43184 | 29.01411 | Mitamba, DRC |
| Katanga |  | 2037 | -7.61878 | 29.78655 | Kyalengwe, Marungu Plateau, DRC |
| Katanga |  | 1973–2030 | -7.70938 | 29.76962 | Pepa, Marungu Plateau, DRC |
| Katanga |  | 1973–2030 | -7.71487 | 29.781 | Pepa, Marungu Plateau, DRC |
| Katanga |  | 1445 | -8.71895 | 27.42265 | Road ca. 17 km west of Mitwaba, DRC |
| Katanga |  | 1568 | -8.6267 | 27.33922 | Mitwaba, DRC |
| Katanga |  | 1428 | -8.46575 | 27.3307 | Mayola River, ca. 3 km east of Kakunko, DRC |
